# Research Review: How to interpret associations between polygenic scores, environmental risks, and phenotypes

**DOI:** 10.1111/jcpp.13607

**Published:** 2022-03-28

**Authors:** Jean‐Baptiste Pingault, Andrea G. Allegrini, Tracy Odigie, Leonard Frach, Jessie R. Baldwin, Frühling Rijsdijk, Frank Dudbridge

**Affiliations:** ^1^ 4919 Division of Psychology and Language Sciences Department of Clinical, Educational and Health Psychology University College London London UK; ^2^ Social, Genetic and Developmental Psychiatry Centre Institute of Psychiatry, Psychology and Neuroscience King’s College London London UK; ^3^ Faculty of Social Sciences Anton de Kom University of Suriname Paramaribo Suriname; ^4^ 4488 Department of Health Sciences University of Leicester Leicester UK

**Keywords:** Polygenic scores, phenotypes, environment, epidemiology, biases

## Abstract

**Background:**

Genetic influences are ubiquitous as virtually all phenotypes and most exposures typically classified as environmental have been found to be heritable. A polygenic score summarises the associations between millions of genetic variants and an outcome in a single value for each individual. Ever lowering costs have enabled the genotyping of many samples relevant to child psychology and psychiatry research, including cohort studies, leading to the proliferation of polygenic score studies. It is tempting to assume that associations detected between polygenic scores and phenotypes in those studies only reflect genetic effects. However, such associations can reflect many pathways (e.g. via environmental mediation) and biases.

**Methods:**

Here, we provide a comprehensive overview of the many reasons why associations between polygenic scores, environmental exposures, and phenotypes exist. We include formal representations of common analyses in polygenic score studies using structural equation modelling. We derive biases, provide illustrative empirical examples and, when possible, mention steps that can be taken to alleviate those biases.

**Results:**

Structural equation models and derivations show the many complexities arising from jointly modelling polygenic scores with environmental exposures and phenotypes. Counter‐intuitive examples include that: (a) associations between polygenic scores and phenotypes may exist even in the absence of direct genetic effects; (b) associations between child polygenic scores and environmental exposures can exist in the absence of evocative/active gene–environment correlations; and (c) adjusting an exposure‐outcome association for a polygenic score can increase rather than decrease bias.

**Conclusions:**

Strikingly, using polygenic scores may, in some cases, lead to more bias than not using them. Appropriately conducting and interpreting polygenic score studies thus requires researchers in child psychology and psychiatry and beyond to be versed in both epidemiological and genetic methods or build on interdisciplinary collaborations.

## Introduction

A polygenic score aims to capture individuals' genetic predispositions for a phenotype. Most often, a weighted sum is computed, with weights obtained from a Genome‐Wide Association Study (GWAS), resulting in a single value per individual and a single variable per sample (e.g. the polygenic score for height). The first (complementary) article of this issue, Allegrini, Baldwin, Barkhuizen, and Pingault (2022), provides a formal definition of polygenic scores, a thorough discussion of the methods available to compute them, and examples of applications in longitudinal settings. Once computed, polygenic scores can be tested for associations with any variable of interest in an independent sample. First, polygenic scores can be associated with their corresponding traits. For example, the polygenic score for height currently explains around 20% of the variance in height (Yengo et al., 2018). Cross‐trait associations can also be tested. For example, the current polygenic score for attention‐deficit hyperactivity disorder (ADHD) not only predicts around 1% of the variance in ADHD symptoms throughout childhood and adolescence but also predicts body mass index (BMI), with some evidence that the link is stronger in adolescence (Liu et al., 2021). Polygenic scores have been shown to be associated with many developmental outcomes. For example, the polygenic score for ADHD is associated with age at walking (Hannigan et al., 2021). Other developmental outcomes predicted by their respective polygenic scores include age at first sexual intercourse or age at first birth (Mills et al., 2021). Intriguingly, polygenic scores for individual traits also predict variables considered to be environmental influences shaping child and adolescent development. For example, a polygenic score for educational attainment computed in children predicts environmental exposures such as maternal education, breastfeeding, or watching television (Krapohl et al., 2017). In addition, polygenic scores for depression, schizophrenia, and neuroticism are associated with being adopted in childhood, while polygenic scores for ADHD, depression, BMI, and intelligence predict exposure to bullying victimisation in early adolescence (Lehto et al., 2020; Schoeler et al., 2019). In sum, polygenic scores are not only used to predict their corresponding phenotype but also related phenotypes, intermediate phenotypes, or environmental exposures (e.g. the polygenic score for schizophrenia predicts schizophrenia but is also associated with substance use, brain structures, and urbanicity) (Newbury et al., 2020). In this review, we discuss the mechanisms that can generate these observed associations and the challenges associated with interpreting them. Several of the challenges of polygenic score research we discuss below, such as measurement error, confounding, mediation, and collider bias, are not specific to genetics but shared with many quantitative sciences, including psychology, psychiatry, and epidemiology. This is because polygenic scores are variables that often share fundamental limitations with other variables typically used in those fields. For example, we may only have inaccurate measures of the phenotype we are interested in, leading to measurement error (e.g. ratings on a questionnaire for ADHD only provide a noisy approximation of the true levels of ADHD symptoms); or the measure we have may not only capture what was intended (e.g. parental ratings of ADHD may also reflect parental biases rather than simply child ADHD).

Because a polygenic score is derived from a linear combination of effect alleles for a given phenotype, it is tempting to assume it captures the genetic liability for this phenotype. Although polygenic scores aim to and can capture genetic effects, several limitations must be acknowledged. First, polygenic scores currently capture only a fraction of SNP‐heritability, which is a measure of heritability due to the effects of all measured common Single Nucleotide Polymorphisms (SNPs) (Campos, Sorensen, & Gianola, 2015; Yang, Zeng, Goddard, Wray, & Visscher, 2017). This gap between the variance captured by polygenic scores and heritability estimates can be conceptualised as measurement error (Pingault, Rijsdijk, et al., 2021; Tucker‐Drob, 2017). For example, the SNP heritability of ADHD is 21.6% but the polygenic score for ADHD explains a maximum of 5.5% of the variance in ADHD (Demontis et al., 2019). This gap is partly due to the imperfect estimation of the associations between SNPs and phenotype in the original GWAS, for example, because of the limited sample size. The resulting noise carries over when the estimates are used as weights in the construction of the polygenic score. As a consequence, the observed null associations between the resulting polygenic scores and outcomes of interest may reflect false negatives (i.e. where a true association exists). Second, statistical biases common to epidemiological studies such as attrition or collider bias can also affect polygenic scores' outcome associations (Akimova, Breen, Brazel, & Mills, 2021). Third, polygenic scores are also afflicted by biases common to many genetic association studies, which are detailed below (Blanc & Berg, 2020). This can lead to false‐positive findings, that is, polygenic scores predicting outcomes in the absence of a true underlying association. Fourth, even when none of these biases are present, the association between polygenic scores and outcomes can be complex to interpret. For example, part of the association between polygenic scores and predicted phenotypes can be mediated (see Figure S1 for an explanation of mediation) by environmental variables (in the same way that genetic effects on lung cancer can occur via smoking) (Gage, Smith, Ware, Flint, & Munafò, 2016; Munafò et al., 2012). Acknowledging this complexity is essential to conduct and interpret polygenic score studies.

Here, we aim to provide a comprehensive overview of factors and biases that can affect associations between polygenic scores, environmental risk, and phenotypes. We provide a formal representation of different issues and how they affect association estimates and interpretation. Where possible, we also provide a brief overview of methods and designs that can be used to deal with those issues.

### Direct genetic effects and measurement error

**Figure 1:**
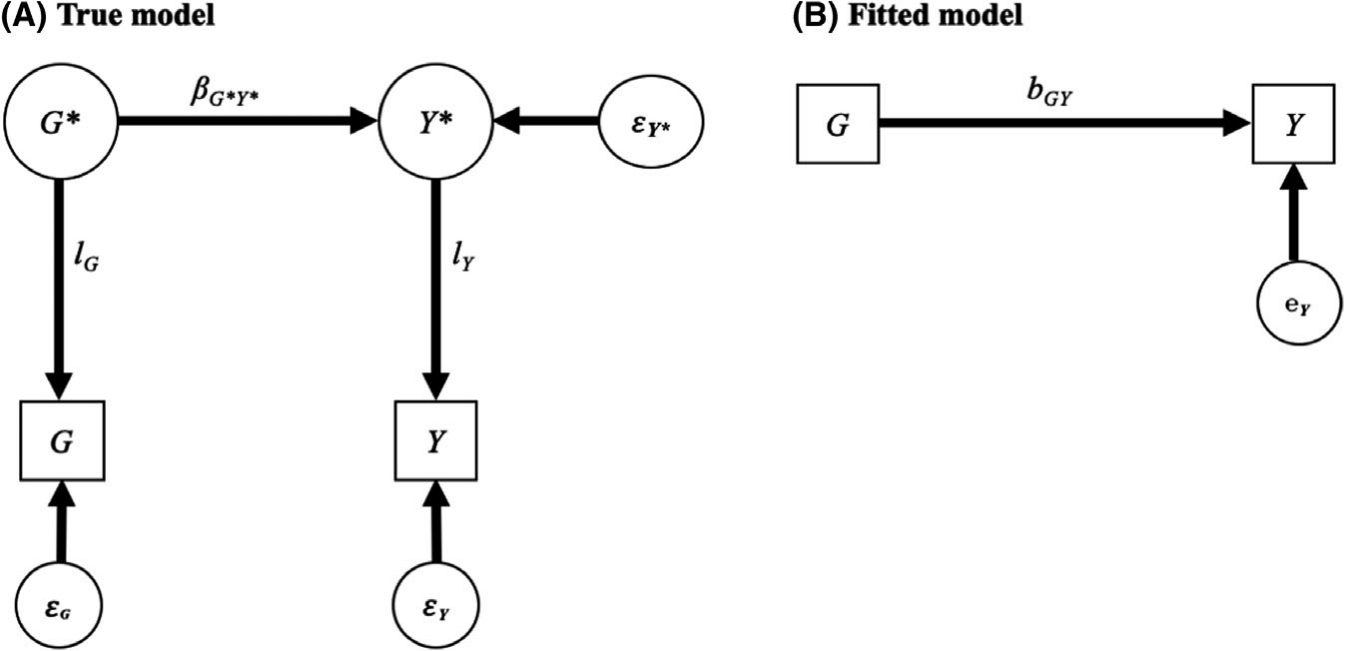
Measurement error. This figure and others present the true model (A) and the fitted model (B). In all figures, latent variables are enclosed within a circle and denoted with * (e.g. Y*). Corresponding measured variables are enclosed within a square (e.g. *Y*). *l* are loadings indexing reliability (e.g. *l_Y_*). *β* stands for beta in the true model (e.g. β_G__*_ _Y__*_), *b* for estimated betas (e.g. *b_GY_*), and *r* for correlation (e.g. *r_GY_*). All betas and loadings are standardised with variances of latent and measured variables equal to 1.

The direct effect of a SNP on a phenotype can be conceived as a causal effect in the sense that a change in the SNP, for example by gene editing, should theoretically lead to a change in the phenotype (Lynch, 2021; Pingault, Richmond, Richmond, & Smith, 2021). Additive heritability captures the addition of all such direct genetic effects. In Figure 1A, direct genetic effects are captured by β_G__*_ _Y__*_, from an additive genetic factor (latent variable G*) to a perfectly measured phenotype (latent variable Y*). In a standardised model, the square of β_G__*_ _Y__*_ equals additive heritability. However, a simple regression of a measured phenotype *Y* on a polygenic score *G* (Figure 1B) does not capture β_G__*_ _Y__*_, because of measurement error. Measurement error can exist for the phenotype (e.g. teacher reports of child anxiety may not reflect the true extent of child anxiety): Y* is imperfectly associated with *Y* (i.e. the standardised loading *l_Y_* from *Y* * to *Y* in Figure 1A, is inferior to 1). In other words, *Y* * explains a limited percentage of the variance in *Y*, that is equal to the reliability *l* ^2^ *_Y_*, resulting in measurement error (1 − *l* ^2^ *_Y_*, i.e. the standardised variance of *Y* minus the reliability). The polygenic score *G* can also be conceived as a measure of the true additive genetic factor with substantial measurement error (G* explains *l* ^2^ *_G_* of the variance in *G*, resulting in measurement error: 1 − *l* ^2^ *_G_*). The fact that polygenic scores are an imperfect measure of the true genetic values has considerable implications for polygenic score studies. As depicted in Figure 1, the fitted parameter of a linear regression of *Y* on *G* (*b_GY_*) is a biased approximation of β_G__*_ _Y__*_. As shown in the Appendix S1, we have:

$$b_{\mathrm{GY}}=l_{Y}l_{G}\beta_{G^{*}Y^{*}}$$

Therefore, *b_GY_* is attenuated as a function of the imperfect measurement of both *Y* * and *G* *. The bias, that is, the fitted parameter minus the true effect is thus:

$$Bias_{\mathrm{GY}}=b_{\mathrm{GY}}‐\beta_{G^{*}Y^{*}}=\beta_{G^{*}Y^{*}}\left(l_{Y}l_{G}‐1\right)$$

If *l_Y_* = *l_G_* = 1, there is no measurement error, and the bias is null. Conversely, if the polygenic score or the outcome is pure noise (*l_Y_* or *l_G_* = 0), then the bias is −β_G__*_ _Y__*_ and the observed association will be null. As a numerical example, consider a highly heritable trait as the outcome, that is,

$$\beta_{G^{*}Y^{*}}^{2}=0.90^{2}=81\%.$$

Assuming a measurement error for *Y* of 20%, that is, 1 − *l* ^2^ *_Y_* = 0.20, so that

$$l_{Y}=\sqrt{0.80}$$

and that G* explains 10% of the variance in *G*, so

$$l_{G}=\sqrt{0.10}.$$

We thus have

$$b_{\mathrm{GY}}=\sqrt{0.80}\times \sqrt{0.10}\times 0.90=0.25.$$

Note that, in Figure 1B, the standardised *b_GY_* = *r_GY_*. The variance explained by the polygenic score in the observed phenotype (*r^2^_GY_*) is therefore close to 6% (0.25^2^), much lower than the underlying heritability. Note that removing measurement error in *Y* increases *b_GY_* but not massively,

$$\sqrt{0.10}\times 0.90=0.28,$$

as the measurement error in the polygenic score is considerably higher. Most current polygenic scores have limited reliability and capture a small percentage of phenotypic variance, sometimes less than 10%, even for highly heritable phenotypes (see Table 1). The reliability of the polygenic score in capturing SNP‐heritability (*h* ^2^_SNP_) can be approximated based on the variance explained by the polygenic score and SNP‐heritability (Table 1 for examples and Appendix S1 for derivation):

$$l_{G}^{2}=\frac{r_{\mathrm{GY}}^{2}}{h_{\text{SNP}}^{2}}$$

**Table 1:** Variance explained by polygenic scores, SNP‐, and twin heritability estimates

Phenotype	*R* ^2^_PGS_	*h* ^2^_SNP_	*R* ^2^_PGS_ /*h* ^2^_SNP_	*h* ^2^_Twin_	References
Educational attainment	0.114	0.147	0.776	0.40	Branigan, McCallum, and Freese (2013); Lee et al., (2018)
Intelligence	0.052	0.190	0.274	0.66	Haworth et al. (2010); Savage et al. (2018)
Childhood intelligence	0.022	0.274^a^	0.080	0.41	Benyamin et al. (2014); Haworth et al. (2010)
Risk taking	0.016	0.045	0.356	0.44	Linnér et al. (2019); Wang, Zheng, Xuan, Chen, and Li (2016)
ADHD	0.055	0.216	0.255	0.76	Demontis et al. (2019); Faraone et al. (2005)
Autism	0.025	0.118	0.212	0.64–0.91	Grove et al. (2019); Tick, Bolton, Happé, Rutter, and Rijsdijk (2016)
Major depression	0.032	0.089	0.360	0.37	Howard et al. (2019); Sullivan, Neale, and Kendler (2000)
Schizophrenia	0.117	0.244	0.480	0.81	Pardiñas et al. (2018); Sullivan, Kendler, and Neale (2003)

For prediction of the PGS in independent cohorts (incremental *R* ^2^ or pseudo *R* ^2^) we provide optimistic values from the original studies, that is, the highest reported *R* ^2^ when multiple replication samples were used and when no pooled *R* ^2^ was provided.

^a^ We calculated the narrow‐sense *h* ^2^_SNP_ on the GWAS summary statistics using LD score regression (Bulik‐Sullivan et al., 2015) to enable comparison with other estimates.

**Figure 2:**
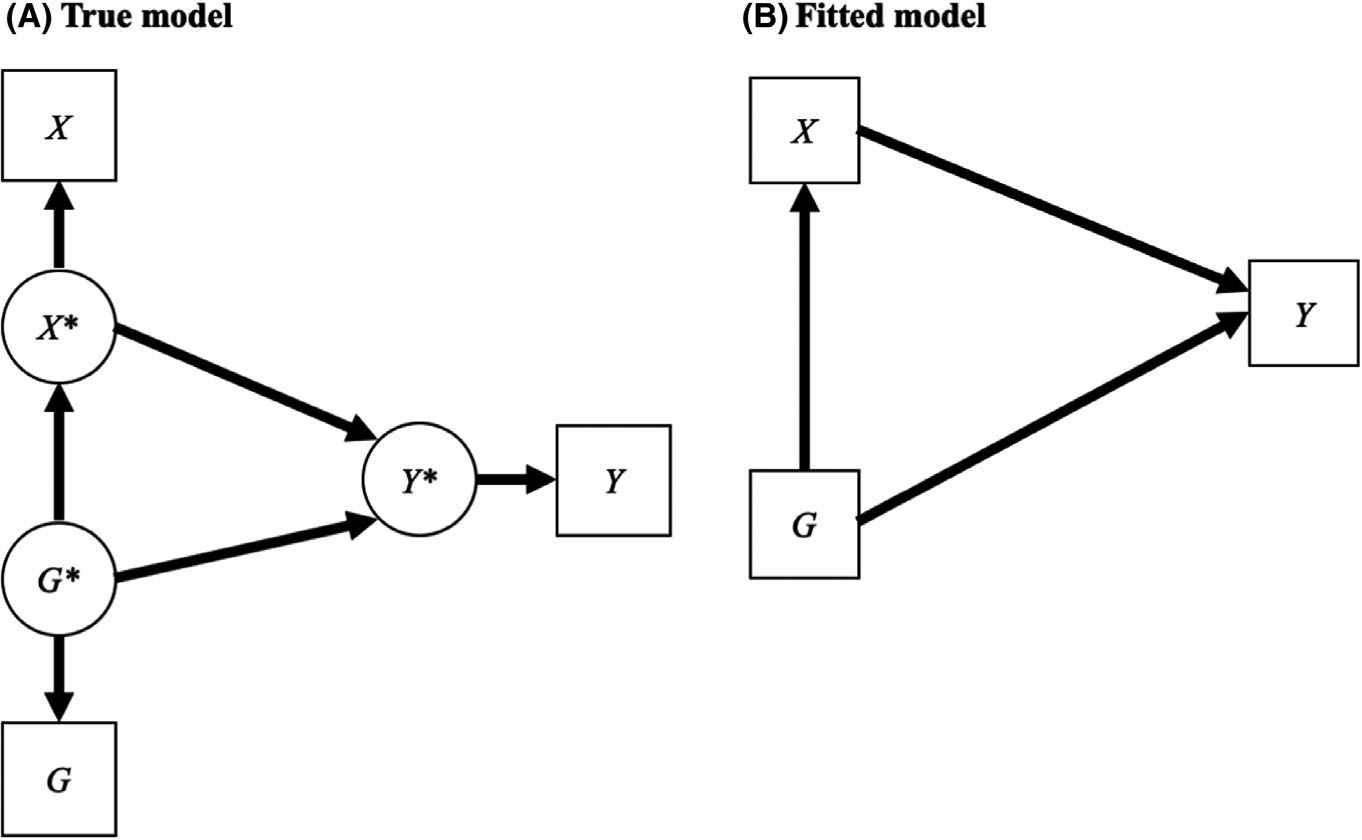
Exposure

### Genetic confounding and mediation

The fact that polygenic scores currently explain a small percentage of (SNP) heritability has wider consequences on models commonly implemented in child and adolescent psychiatry and beyond. Let us extend the previous model by adding a risk factor *X* (Figure 2). Figure 2A illustrates genetic confounding where a genetic factor (G*) confounds the true association between the exposure (*X*  *) and the outcome (*Y* *) (see Figure S1 for confounder definition). The true genetic confounding effect is equal to β_G__*_ _X__*_ β_G__*_ _Y__*_ (Pingault, Rijsdijk, et al., 2021). Genetic confounding is pervasive in psychiatric research. This is because, in addition to influencing psychiatric outcomes, genetic factors are associated with many exposures typically classified as environmental, thereby generating confounding. For example, the polygenic score for schizophrenia has been found to be associated with urbanicity, which is a known exposure for schizophrenia. Polygenic scores have been associated with many environmental exposures relevant to child and adolescent psychology and psychiatry, including maternal education, parenting, breastfeeding, or bullying victimisation (Krapohl et al., 2017; Newbury et al., 2020; Schoeler et al., 2019). Recognising the role of genetics in confounding associations between exposures and outcomes, researchers have recently started to adjust for polygenic scores when examining the association between a risk factor and an outcome (Croft et al., 2019; Lee et al., 2021; Paul et al., 2021). For example, Croft et al. investigated the association between trauma and later psychotic experience, adjusting for a number of confounders including polygenic scores for schizophrenia and bipolar disorders. However, because those polygenic scores have low reliability, the adjustment is incomplete and can lead to the incorrect conclusion that the risk factor is still associated with the outcome after adjusting for genetic confounding. For simplicity, we assume that *X* * and *Y* * are perfectly measured and we compute the fitted parameter b_X__*_ _Y__*_ when adjusting for the observed polygenic score. We show in the Appendix S2 that:

$$b_{X^{*}Y^{*}}=\beta_{X^{*}Y^{*}}+\frac{\beta_{G^{*}X^{*}}\beta_{G^{*}Y^{*}}\left(1‐l_{G}^{2}\right)}{1‐l_{G}^{2}\beta_{G^{*}X^{*}}^{2}}$$

where the fitted parameter b_X__*_ _Y__*_ consists of the true beta β_X__*_ _Y__*_ plus the true genetic confounding effect but scaled by the measurement error of *G* (1 − *l* ^2^ *_G_*) (Pingault, Rijsdijk, et al., 2021). The fitted beta will thus be larger than the true beta as genetic confounding has not been entirely adjusted for, to the extent that *G* is a noisy measure of G*. This means that even when the true beta is zero and X* has no effect on Y*, the observed beta will not be null. The bias:

$$Bias_{X^{*}Y^{*}}=\frac{\beta_{G^{*}X^{*}}\beta_{G^{*}Y^{*}}\left(1‐l_{G}^{2}\right)}{1‐l_{G}^{2}\beta_{G^{*}X^{*}}^{2}}$$

can be almost as large as the true genetic confounding effect if the reliability of *G* (*l* ^2^ *_G_*) is low (corresponding to high measurement error), which is often the case. Let’s take the example of (i) an exposure X* with no effect on the outcome Y*, that is,

$$\beta_{X^{*}Y^{*}}=0.$$

(ii) a heritable outcome Y* with a SNP‐heritability

$$h_{\text{SNP}}^{2}=0.30,$$

leading to

$$\beta_{G^{*}Y^{*}}=\sqrt{0.30},$$

as there are no genetic effects on Y* mediated by the exposure. We also have substantial genetic effects on the exposure, that is,

$$\beta_{G^{*}X^{*}}=\sqrt{0.15}$$

and a polygenic score that captures 3% of the variance in Y*. We thus have:

$$l_{G}^{2}=\frac{0.03}{0.30}=0.10$$

and a bias of

$$Bias=\frac{\sqrt{0.15}\times \sqrt{0.30}\times \left(1‐0.10\right)}{\left(1‐0.1\times 0.15\right)}=0.19.$$

Consequently, with a true effect that is null, we have a fitted beta of 0.19 despite adjusting for the polygenic score. As such, even substantial associations like the one mentioned above between trauma and later psychotic symptoms can be theoretically explained by genetic confounding that remains even after adjusting for polygenic scores.

A related question of interest, also represented in Figure 2, is how much of the effect of the polygenic score is mediated by the risk factor, that is, the pathway G*→X*→Y*. Several studies have aimed to examine to what extent the effect of a polygenic score on an outcome is mediated by important risk factors throughout development (Belsky et al., 2016; Wertz et al., 2019). For example, Belsky et al. found that the association between the polygenic score for education and socioeconomic outcomes was mediated by child characteristics such as cognitive ability, self‐control, and interpersonal skills. Importantly, Figure 2 illustrates the ambiguity of the term ‘direct effect’ as the effect of G* is now mediated (indirect) via *X*. In a sense, all genetic effects are indirect, whether mediated by physiological pathways or environmental risk factors. To add to the confusion, in mediation analyses, the term direct effect has a standard meaning as the effect that is not mediated by the mediator(s) being modelled. The term ‘direct genetic effect’ defined above thus only refers to the fact that the individual phenotype Y* is ultimately explained by the individual genetic factor G* without the additional biases discussed below, and does not preclude mediation. We come back to this issue and propose a new terminology below.

The true mediated effect is obtained by multiplying betas along the path from G* to Y*, that is,

$$\beta_{M}=\beta_{G^{*}X^{*}}\beta_{X^{*}Y^{*}}$$

The fitted mediated effect using the polygenic score is (as shown in the Appendix S2):

$$b_{M}=l_{G}\beta_{M}+l_{G}\beta_{G^{*}X^{*}}Bias_{X^{*}Y^{*}}$$

The fitted mediated effect is therefore the true mediated effect scaled by *l_G_* (i.e. *l_G_β_M_*) but there is also an additional term corresponding to an additional ‘mediation’ path via the bias in X*Y*. This is because, as we have shown above, the fitted b_X__*_ _Y__*_ is under‐corrected for genetic confounding. This undercorrection leads to an overestimation of b_X__*_ _Y__*_ which, in turn, leads to an overestimation of the fitted mediation effect. The counter‐intuitive consequence is that, when using a polygenic score, the proportion of genetic effects mediated by a given risk factor *X* is over – rather than underestimated. The estimated proportion mediated is:

$$p_{M}=\pi_{M}+\frac{Bias_{X^{*}Y^{*}}r_{G^{*}X^{*}}}{r_{G^{*}Y^{*}}},$$

where *π_M_* is the true proportion mediated, to which an additional proportion is added that includes the additional ‘mediation’ path via the bias in X*Y* in the numerator and the true total association between the genetic factor and the outcomes in the denominator (r_G__*_ _Y__*_). It is therefore possible to have a nonnull mediated effect when using the polygenic score even if the true percentage is null (i.e. a false positive). In the example above, because the study used a polygenic score for educational attainment, which was not entirely reliable, the path from child self‐control to later socioeconomic outcomes is likely under‐corrected for genetic confounding and thus exaggerated. In turn, the fitted mediated effect from the polygenic score for education to adult socioeconomic outcomes via child self‐control is likely exaggerated.

Recently, we have proposed a method to account to some extent for the low reliability of polygenic scores based on external estimates of heritability (Pingault, Rijsdijk, et al., 2021). Using structural equation modelling, we can correct for measurement error by including the polygenic score as an imperfect measure of additive genetic factors as in Figure 2B. This is akin to a sensitivity analysis where we consider what would happen if we had measured a polygenic score capturing additive heritability. An advantage of this genetic sensitivity analysis over other sensitivity analyses of unmeasured confounders is that we have an external measure of the importance of this unmeasured factor, using estimates such as *h* ^2^_SNP_ or even additive heritability measured from twin studies.

### Collider bias

**Figure 3:**
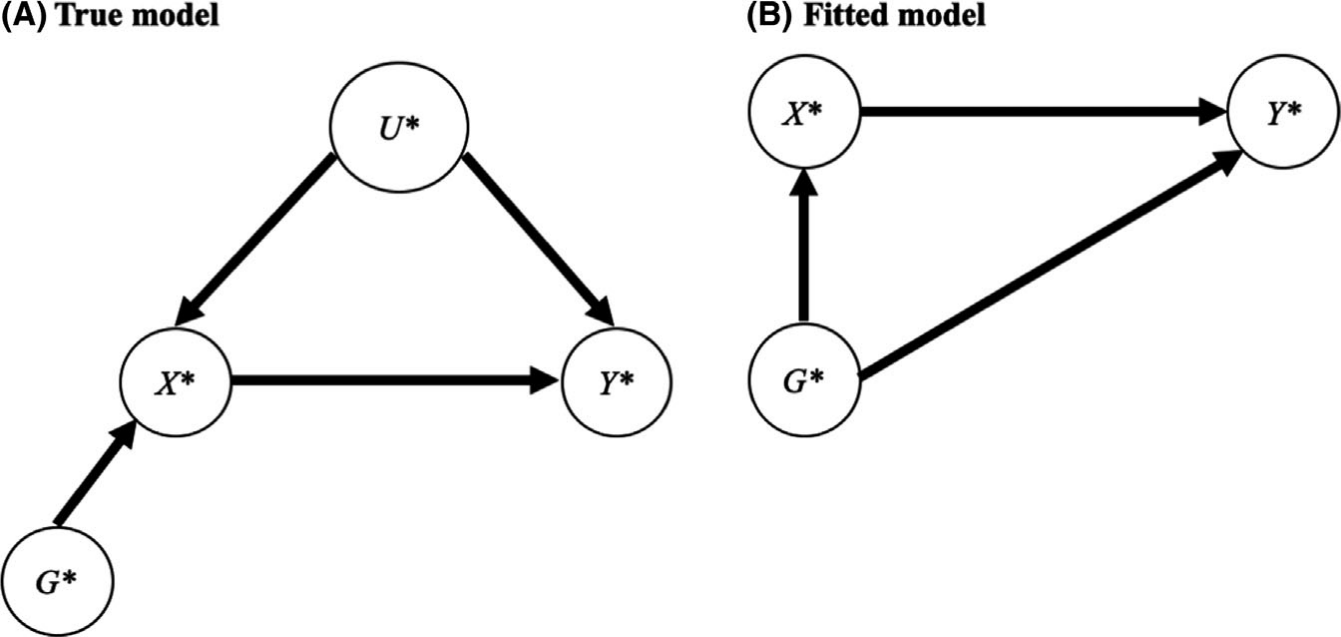
Collider bias

Collider bias, that is, the bias incurred from adjusting for a collider should also be considered when adjusting for polygenic scores. In Figure 3A, X* is a collider for G* and U* as the two arrows from the predictors G* and U* are directed to X*, that is, they collide in X*. To illustrate collider bias, let us take the example of two predictors of referral for treatment of depression in adolescents, which thus becomes a collider. The first predictor is a dichotomised polygenic score (high vs. low risk) and the second is a dichotomised environmental risk (high vs. low risk). Let us assume for simplicity that the two are independent and that being a high risk on either factor necessarily leads to referral. Because the two are independent in the population, knowing that one individual is at high environmental risk gives no information on risk status for the polygenic score (i.e. the two predictors are not associated). However, within the referred adolescents only (i.e. one stratum of the outcome), the two become strongly negatively associated. This is because we know that for an adolescent to be referred they have to be high risk on either or both variables. Therefore, if we know that a referred adolescent has a low‐risk polygenic score, we also know with certainty that they must be at high environmental risk. Polygenic and environmental risks become associated by adjusting for the collider (the adjustment is represented here by stratifying for the collider when testing for the association in referred adolescents only). Similarly, collider bias can generate spurious associations between genetic variables (variants or polygenic scores) and environmental risk factors if analyses are restricted to cases of a disease.

More generally, adjusting for a collider can induce an association between independent predictors of the collider, which can generate a knock‐on bias in the model. In Figure 3A, U* stands for unknown (or unmeasured) nongenetic confounders of the association between X* and *Y* *. The fitted model, Figure 3B, therefore does not include U* (to simplify, we first assume that G*, X*, and Y* are measured). Importantly, G* is not a confounder here as it causes X* but does not cause Y* directly. In theory, adjusting for G* should not modify the association between X* and *Y* *. In practice, in a model regressing *Y* * on both X* (the collider) and G*, the association between X* and *Y* * is biased. This specific case has been termed bias amplification as the bias can be so severe that the adjusted estimate of X*→*Y* * is further from the causal effect than the unadjusted correlation between X* and *Y* * (Myers et al., 2011). As shown by others, the estimated adjusted path of the association between the genetic factor and the outcome is

$$b_{G^{*}Y^{*}}=\beta_{G^{*}Y^{*}}‐\frac{\beta_{G^{*}X^{*}}\beta_{U^{*}X^{*}}\beta_{U^{*}Y^{*}}}{1‐\beta_{G^{*}X^{*}}^{2}}$$

(Akimova et al., 2021). The numerator of the second term (bias) is akin to an indirect path *G* *→*X* *←*U* *→Y * in Figure 3A. Note that this path is normally not a legitimate backdoor path as it is blocked in X*; adjusting for the collider X* unblocks this path and the association between G* and U* (see also Figure S1 for an illustration of concepts such as backdoor path and blocked path). If all paths in the model are positive, the adjusted effect of the genetic factor on the outcome will be underestimated. In turn, we have

$$b_{X^{*}Y^{*}}=\beta_{X^{*}Y^{*}}+\frac{\beta_{U^{*}X^{*}}\beta_{U^{*}Y^{*}}}{1‐\beta_{G^{*}X^{*}}^{2}}$$

Note that β_X__*_ _Y__*_ + β_U__*_ _X__*_ β_U__*_ _Y__*_ would correspond to the true residual association between X* and *Y* * once G* has been adjusted for, that is, the association free of genetic confounding (because U* is not measured, the residual association is equal to the causal effect plus the backdoor path via U*). The bias thus comes from the denominator of the second term, which is equal to the standardised variance of X* (1) that is not explained by the effect of G* on the exposure X* (*β* ^2^ _G__*_ _X__*_). As such, the more variance G* explains in X* the smaller the denominator will be and the larger the bias. In other words, once the effect of G* is removed, *U* * explains a larger proportion of the variance in *X* *, which magnifies the bias via U* (Myers et al., 2011). Concretely, this means that adjusting for the polygenic score corresponding to the exposure (thus explaining more variance in the exposure), should be avoided, as it might increase the bias rather than remove genetic confounding. Instead, the adjustment should be based on the polygenic score for the outcome (Pingault, Rijsdijk, et al., 2021).

When allowing for measurement error in *G*, *X*, and *Y* and a collider bias as represented in Figure S2, the fitted *b_XY_* between observed variables becomes more complex (see Appendix S3 for derivation and detailed explanations). Instead of an unbiased scenario where *b_XY_* = β_X__*_ _Y__*_, we have:

$$b_{\mathrm{XY}}=\frac{l_{X}l_{Y}\left[\beta_{X^{*}Y^{*}}\left(1‐l_{G}^{2}\beta_{G^{*}X^{*}}^{2}\right)+\beta_{U^{*}X^{*}}\beta_{U^{*}Y^{*}}+\beta_{G^{*}X^{*}}\beta_{G^{*}Y^{*}}\left(1‐l_{G}^{2}\right)\right]}{1‐{\left(l_{G}l_{X}\beta_{G^{*}X^{*}}\right)}^{2}}$$

We note several components to this expression. Reliability terms are present in both the numerator and the denominator. Note that this expression simplifies to the expression above when *l_G_* = *l_X_* = *l_Y_* = 1. The second term of the numerator (β_U__*_ _X__*_ β_U__*_ _Y__*_) refers to the collider bias and is similar to what we obtained in the simpler expression of b_X__*_ b_Y__*_ above. The third term includes β_G__*_ _X__*_ β_G__*_ _Y__*_ and refers to the genetic confounding effect that remains unadjusted because of the measurement error in *G* (1 − *l* ^2^ *_G_*). Taken together, these different components mean that, as polygenic scores become more accurate (increasing *l_G_*), genetic confounding should be better adjusted for while the collider bias will worsen. The complexity of this expression also demonstrates why simply adjusting for a polygenic score does not adjust for genetic confounding.

### Interpreting genetic associations with environmental risk

As noted in the introduction, polygenic scores have been associated with many variables that are typically considered environmental, such as maltreatment, parenting, and bullying. This is typically referred to as gene–environment correlation (abbreviated *r_GE_*), which can be active/evocative, discussed in this section or passive, discussed later.

**Figure 4:**
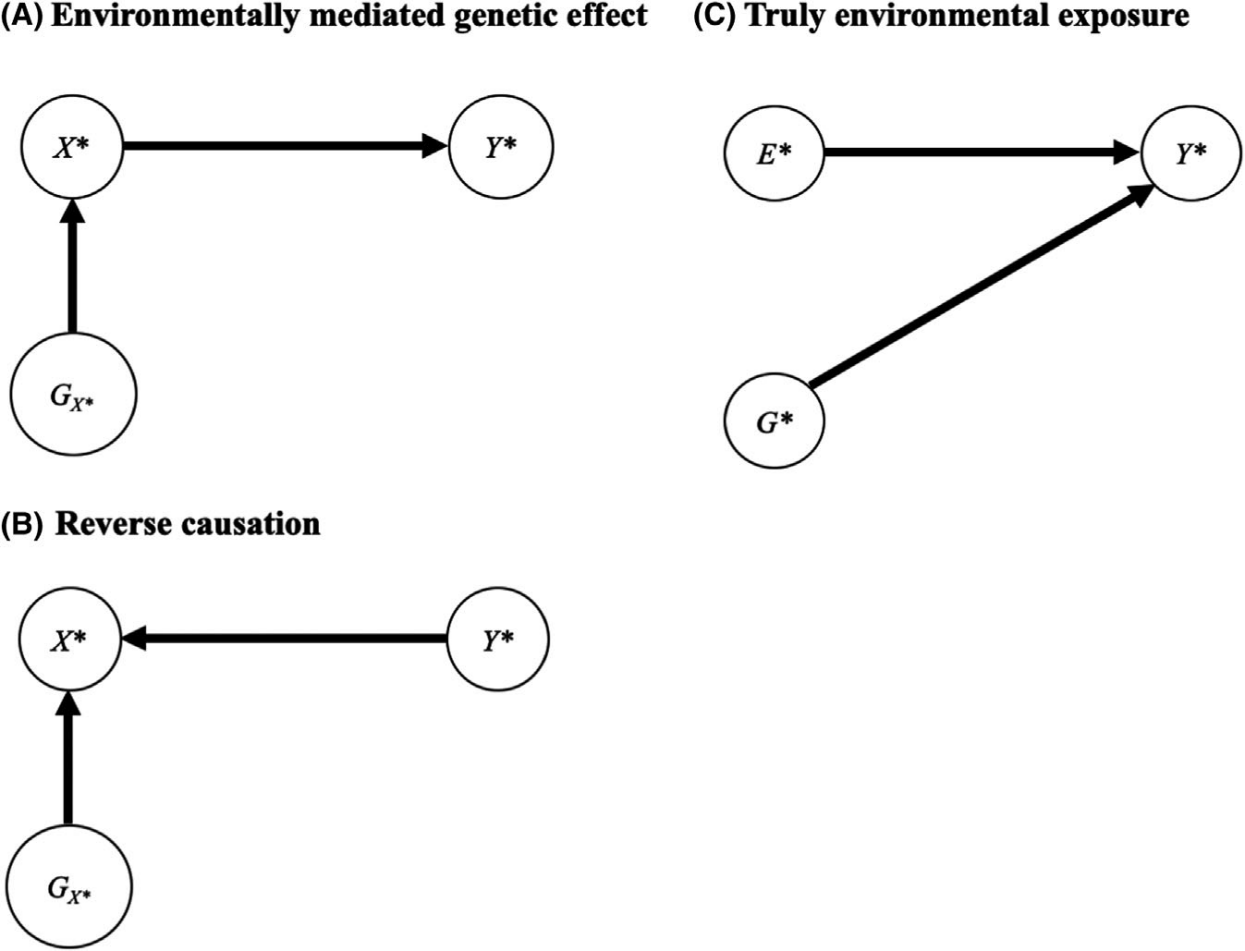
Environmental risk

Figure 4A shows that the effect of a genetic variant (or a polygenic score) for X* denoted G_X__*_ has an indirect effect on Y* to the extent that X* causes Y*. This means that, when conducting a GWAS of *X* *, the same genetic variant will be associated with *Y* * with an effect of β_G__*_ _X__*_ β_X__*_ _Y__*_. When the causal effect is strong, the signal in the GWAS of *Y* * can also be strong, for example, some genetic variants significantly associated with lung cancer are, in effect, genetic variants that directly explain smoking (smoking mediates the effect of those genetic variants on lung cancer) (Gage et al., 2016; Munafò et al., 2012). Similarly, a polygenic score for ADHD was associated with experiencing bullying victimisation. Children with a high ADHD polygenic score are more likely to develop impulsivity and hyperactivity, which, in turn, may evoke harsher reactions from their peers. In the same study, the polygenic score for BMI was also associated with experiencing bullying. The genetic liability to BMI leads to higher BMI, which, in turn, is a known risk factor for experiencing bullying (this also illustrates that such associations may vary according to context, depending for example on the social perception of high BMI). These are examples of evocative gene–environment correlations when genetically influenced characteristics (impulsivity‐hyperactivity or BMI) ‘evoke’ a particular environmental response. Active gene–environment correlations are conceptually distinct in that the child is said to actively shape their own environment (e.g. by selecting a more turbulent peer group) (Plomin, DeFries, Knopik, & Neiderhiser, 2013).

More generally, when an exposure causes an outcome, exposure‐related genetic variants will be captured in the outcome GWAS (Gage et al., 2016). As such, with sufficient power, a polygenic score for any given phenotype will necessarily be associated with heritable environmental exposures for this phenotype. This may lead to the conclusion that polygenic score associations with disease exposures tautologically reflect the epidemiology of the disease, in the sense that the polygenic score for *Y* * will be associated with every variable associated with *Y* *. However, even when X* and Y* are associated at the phenotypic level, genetic variants (or a polygenic score) for *Y* * need not be associated with X* in case of reverse causation (Figure 4B) or of a purely environmental (nonheritable) exposure such as exposure to an earthquake (Figure 4C).

The fact that genetic effects on the outcome reflect the causal effect of heritable exposures is central in genetically informed causal inference methods such as Mendelian randomisation (i.e. if X* causes *Y* *, then any genetic variant associated with X* will necessarily be associated with *Y* via the mediation pathway G_X__*_→X*→*Y* *) (Richmond & Smith, 2021). G_X__*_ is called an instrument for *X* * as it is used to calculate the causal effect of X* on Y*. Polygenic scores can also, to some extent, be used as proxies of exposures (Schoeler et al., 2019), and can, under strict assumptions, provide causal effects that are mathematically equivalent to effects obtained in Mendelian randomisation analyses (Dudbridge, 2021).

## Perception of environmental risk

**Figure 5:**
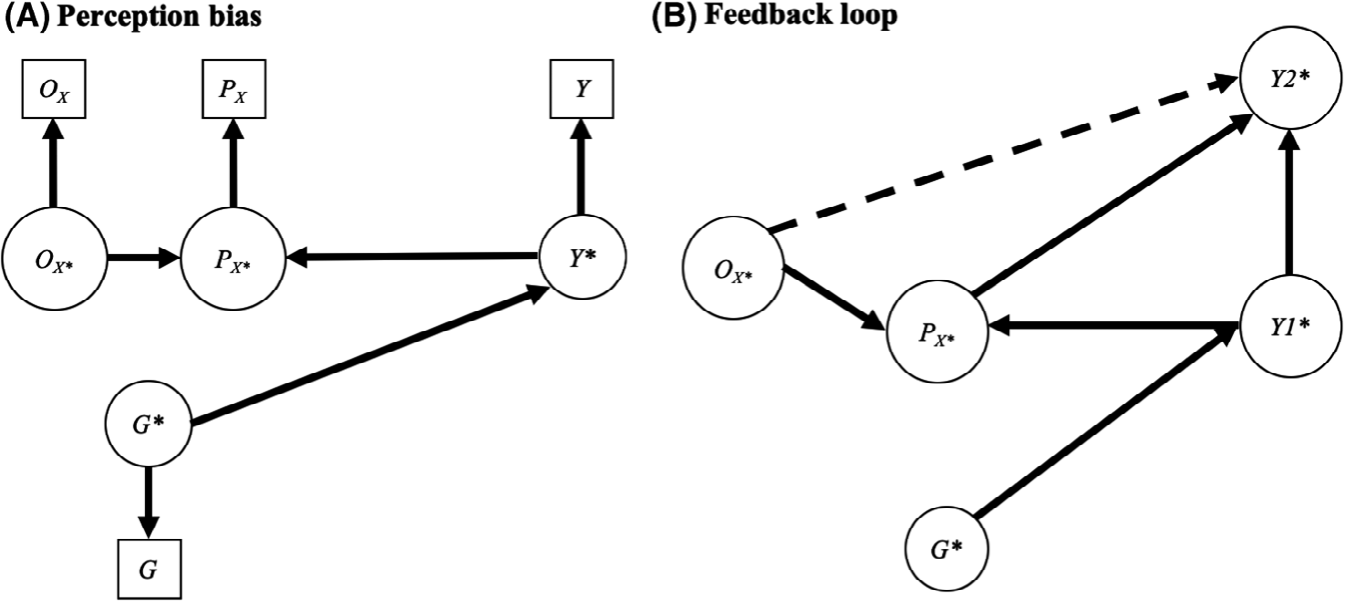
Risk perception

The rating of many psychopathological symptoms – such as low mood – is intrinsically subjective, relying on an individual’s self‐report. This not only leads to complex measurement issues but also affects aetiological research. This is because the subjective appraisal of internal states extends to the perception of external environments. Perception biases susceptible to affect risk appraisals, such as paranoia, delusions, or negative cognitions, are common features of many psychiatric disorders. Interpreting genetic associations with environmental exposures are therefore more complex when the environmental measure involves perception (e.g. it is self‐reported). For example, we could observe an association between a polygenic score for schizophrenia and self‐reported neighbourhood violence: (a) without any path from the polygenic score to objective levels of neighbourhood violence (i.e. no genuine gene–environment correlation between the genetic factor and the environmental exposure); and (b) without any role of neighbourhood violence in the aetiology of schizophrenia. This is illustrated in Figure 5A, where an objective risk (O_X__*_, e.g. actual neighbourhood violence) informs the perception of the risk (P_X__*_, e.g. perceived neighbourhood violence). Risk perception is also influenced by a phenotype Y* (e.g. schizophrenia symptoms) itself influenced by genetic factors (G*). This will lead to observed correlations between *G* and *P_X_* (false positive *r_GE_*) but not between *G* and *O_X_*. The objective risk factor *O_X_* is also unrelated to the phenotype *Y*. The observed correlations between *G*, *P_X_*, and *Y* do not imply that the polygenic score has an environmentally mediated effect; rather, the polygenic score influences the outcome (e.g. schizophrenia symptoms) which leads to perceived environmental risk. Even if measures of perceived and objective environmental exposures are associated (Figure 5A), the potential for genetically influenced perception bias means that measures of perceived risk cannot be used to demonstrate *r_GE_* or causal effects of objective exposures on outcomes.

However, the perceived environmental risk might also feed into later psychiatric symptoms, as illustrated in Figure 5B. For example, perceived neighbourhood violence (whether consistent or not with objective levels of violence) could lead to later paranoid symptoms (i.e. Y2* in Figure 5B). In this case, perception of environmental risk itself becomes important in the aetiology of psychopathology. Notably, objective risk also affects psychopathology, but these effects are completely mediated via the perception of risk (dashed arrow from O_X__*_ to Y 1* indicating an absence of direct effect of O_X__*_ after accounting for P_X__*_). This mediation scenario might explain evidence showing that objective measures of adverse childhood experiences (such as maltreatment or bullying) are not associated with psychopathology after accounting for perceptions of these experiences (Baldwin & Degli Esposti, 2021; Danese & Widom, 2020). In this situation, intervening on the objective risk factor would lead to a subsequent reduction in psychiatric symptoms (Y2*). But interventions targeting the perception itself could be beneficial when intervening on the objective exposure is complex or impossible (e.g. when an adult has been exposed to early adversity). Note that genetic effects can also tag perception processes (i.e. a polygenic score for Y2* would reflect the path G*→Y1*→P_X__*_→Y2*).

## Additional biases arise from jointly modelling environmental risk and polygenic scores

Selection bias and attrition – that is, the biases resulting from a nonrandom (self‐) selection or retention into the sample – can be considered as special cases of collider bias (Munafò, Tilling, Taylor, Evans, & Davey Smith, 2018). When polygenic scores and measures of environmental risk independently predict participation or retention in a study sample, spurious associations between the polygenic scores and measures of environmental risk can be generated. This is because restricting the analysis to (the remaining) participants is equivalent to stratifying on the collider.

Estimating gene–environment interactions using single genetic variants has been hopeless (Duncan & Keller, 2011). While there is a renewed interest in estimating gene–environment interactions using polygenic scores, the aforementioned biases still apply. Measurement error can lead to underestimating the interaction term. The presence of *r_GE_* can lead to spurious gene–environment interactions (Dudbridge & Fletcher, 2014). Furthermore, collider bias also changes gene–environment interactions in nonintuitive ways (Akimova et al., 2021).

### Demographic biases

**Figure 6:**
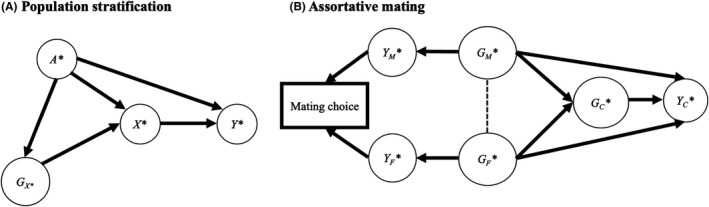
Demographic bias. Adjustment for mating choice is represented by the thick square around the variable in 7B. This creates the co‐path (dashed line) between genetic factors for mothers (*G_M_* *) and fathers (*G_F_* *). In turn, this affects downstream analyses. For example, the variance in the child outcome Y_C__*_ now includes an additional component via the co‐path between *G_M_* * and *G_F_* *. Note that assortative mating can lead to cross‐trait associations, for example between height and education. At the SNP level, assortative mating can thus lead to correlations (in the child) of SNPs that should be uncorrelated, for example, an SNP for height in one chromosome and an SNP for education in another

Bias from population stratification arises when ancestry confounds the relationships between polygenic scores, environmental risk factors, and outcomes (Figure 6A). The chopstick example is a classical illustration of biases arising from population stratification (Hamer & Sirota, 2000). With two populations of different ancestries, one eating with chopsticks, the other with forks, a GWAS of chopstick eating will uncover many genetic variants (i.e. all alleles with different frequencies between the two groups). Such variants do not indicate the discovery of chopstick eating genes but are markers of ancestry. Many factors, including the geographic location of populations, language, or religion can be influenced by ancestry, potentially leading to false positives in GWAS and downstream investigations including polygenic score studies. Population stratification is typically accounted for by controlling for principal components that reflect ancestry (Uffelmann et al., 2021). However, population stratification can be granular and hard to fully capture, arising for example from movements of subpopulations within a region over centuries (Young, Benonisdottir, Przeworski, & Kong, 2019). Within family, analyses can be useful to control for residual population stratification. For example, full biological siblings share the same parents and hence the same ancestry. A within‐sibship GWAS can examine the role of inheriting a risk variant by comparing outcomes in a sibling who inherited the variant versus a sibling who did not (Howe et al., 2021).

The genetic relatedness between first‐degree relatives, such as a parent and their biological offspring, implies that the correlation between their polygenic scores for any given trait should be .50. Conversely, polygenic scores of genetically unrelated people, for example, the two parents, should be uncorrelated. However, a nonzero correlation can be observed between the two parents due to assortative mating – that is, the fact that people choose their partner based on some heritable phenotypic characteristics such as education. This mating choice can be construed as a collider (Figure 6B). Stratifying for this collider by conducting within family analyses leads to intraclass correlations, for example, the polygenic score of the mother is correlated positively with the polygenic score of the father. Assortative mating leads to a number of possible biases in downstream analyses involving the offspring (Figure 6B).

### Genetic nurture

**Figure 7:**
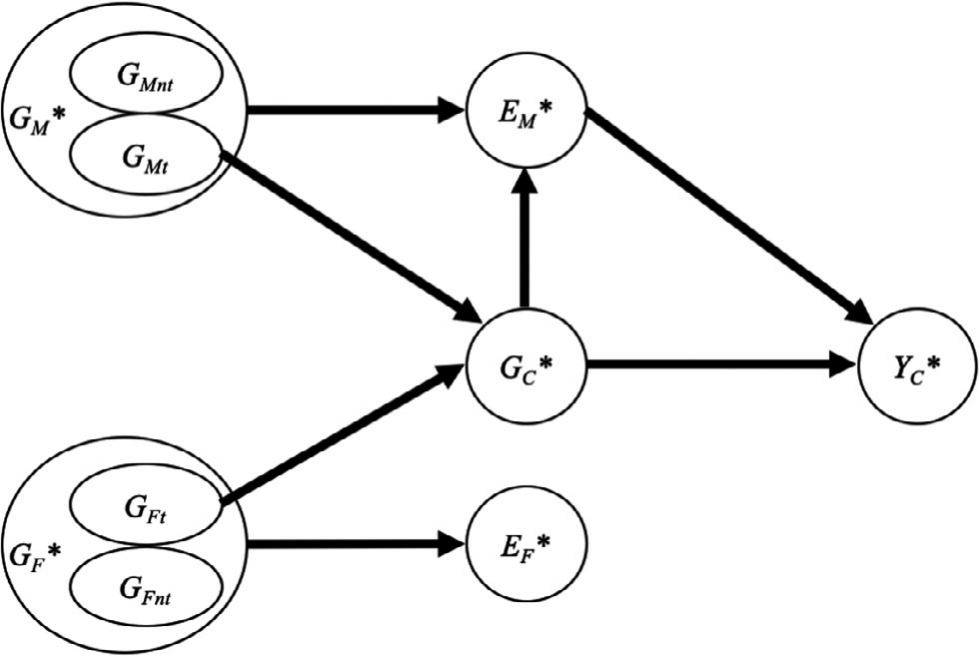
Genetic nurture. Genetic nurture (or familial genetic effects) occurs when parental genetics influence offspring outcomes via environmental pathways, for example, *G_M_* *→*E_M_* *→*Y_C_* * for mothers. Note that even transmitted alleles (*G_Mt_* & *G_Ft_*) can have a genetic nurture effect as, being part of the parental genome, they affect the environment to the same extent as the nontransmitted alleles. By contrast, genetic transmission arises from the fact that child genetics (*G_C_* *) comprises maternally (*G_Mt_*) and paternally (*G_Ft_*) transmitted genetic material, leading to the paths *G_Mt_* (*G_Ft_*)→*G_C_* *→*Y_C_* *. Direct genetics effects (or individual genetic effects) are the effects originating in the child genome (*G_C_* *→*Y_C_* *), free of inflation arising from familial genetic effects. Passive gene–environment correlation refers to the fact that the child genetics can be correlated to the child's environment because of the backdoor path via parental genetics, for example, *G_C_* *←*G_M_* *→*E_M_* *. In addition, there can be an active/evocative gene–environment correlation (*G_C_* *→*E_M_* *). In the figure, no active/evocative gene–environment effect is present on the father’s side (no direct arrow from *G_C_* * to *E_F_* *). But note that a correlation would still be observed between G_C__*_ and E_F__*_ because of the passive gene–environment correlation (*G_C_* *←*G_F_* *→*E_F_* *). The association between E_M__*_ and Y_C__*_ is genetically confounded by paths *Y_C_* *←*G_C_* *←*G_M_* *→*E_M_* * (passive) and *Y_C_* *←*G_C_* *→*E_M_* * (active/evocative). Similarly, a correlation between *E_F_* * and *Y_C_* * would be observed despite an absence of an effect in the figure from *E_F_* * to *Y_C_* *. Finally, dynastic effects refer to the backdoor path *G_C_* *←*G_M_* *→*E_M_* *→*Y_C_* *. Dynastic effects imply that a correlation can be observed between *G_C_* * and *Y_C_* * even if there were no individual genetic effects (no path *G_C_* *→*Y_C_* *). Dynastic effects lead to biased association estimates obtained from GWAS and downstream analyses including polygenic score and Mendelian randomisation analyses. Note that genetic nurture can be more simply represented as population stratification in Figure 6A, by replacing A* with parental genetics. Figure 7 contains several simplifications: (a) only latent variables are represented; (b) the absence of correlation between *G_M_* * and *G_F_* *, that is, no assortative mating; (c) no representation of the parental phenotype (e.g. *G_M_* * it is likely to influence parental phenotypes like depression, which, in turn, influence the environment *E_M_* *); (d) only one path is represented between parental genes and child outcomes whereas many parental phenotypes and environmental variables are likely to explain the relationship; and (e) no correlation is represented between *E_M_* * and *E_F_* * which could partially or totally overlap

The term genetic nurture may sound like an oxymoron but is one of the complex interplays between nature and nurture. As mentioned above, some genetic effects can be mediated by environmental factors. Genetic nurture effects are similar within an intergenerational context, that is, parental genetics influence child outcomes via environmental pathways. For example, parental genetic variants, even when not transmitted to the child, still influence parental depression, which in turn may affect parenting, which in turn can affect child internalising problems (Cheesman et al., 2020). In other words, genetic factors in the parents, even when not transmitted to the child, can affect the child’s outcomes by affecting the way that parents nurture the child. This is depicted in Figure 7, which also aims to clarify a number of related concepts arising from the fact that children inherit both genetic variants and environments from their parents, including passive and active/evocative gene–environment correlations, direct genetic effects, dynastic effects, and genetic confounding. All these concepts are particularly important in child and adolescent psychology and psychiatry where a key endeavour is to understand the role of parents in shaping child development.

Note that the concept of indirect genetic effects is sometimes preferred to genetic nurture. This is partly because it complements the concept of direct genetic effects. Most importantly, genetic nurture seems to imply that the effects involve nurturing from parents, which is not necessarily the case. For example, genetic variants associated with increased education in parents may affect child outcomes via nurturing behaviours (e.g. reading to the child) or other indirect mechanisms (e.g. the school that the child is sent to). However, as highlighted above, the terms direct and indirect genetic effects are also unsatisfactory as (i) all genetic effects are indirect via physiological and environmental pathways and (ii) confusion arises with standard terminology used in mediation analyses when assessing whether either direct or indirect genetic effects are mediated by intermediate variables. We therefore propose to replace direct genetic effects with individual genetic effects defined as effects that originate in the individual genome. And to replace indirect genetic effects with familial genetic effects defined as effects that originate in the genome of family members, such as parents or siblings, independent of genetic transmission (see Figure 7). These terms have the advantage of not referring to either causality, pathways (direct/indirect), or mechanisms (nurture) but simply describe in whom the genetic effects originate from. Causal individual genetic effects or causal familial genetic effects occur when those effects are free from biases (e.g. assortative mating).

Figure 7 and caption explain a number of challenges arising from the combination of familial and individual genetic effects in polygenic score studies: (a) the unadjusted association between child polygenic scores and child outcomes also captures familial genetic effects rather than just individual genetic effects, for example, the association between a child’s polygenic score for education and their own educational achievement is inflated by environmentally mediated familial genetic effects (Wang et al., 2021); (b) child polygenic scores can be associated with environmental exposures in the absence of evocative gene–environment correlation, for example, it is possible that the child polygenic score for hyperactivity–impulsivity is associated with parenting not because those genetically influenced traits evoke harsher parenting (corresponding to evocative *r_GE_*) but because hyperactivity–impulsivity in the parents impacts their parenting (leading to passive *r_GE_*); and (c) environmental exposures and child outcomes can be associated in the absence of a causal effect of the environment (e.g. trauma can be associated with later psychotic symptoms in the absence of a causal effect of trauma).

Note that family‐based studies are starting to address these challenges. As mentioned above, sibship GWAS can better estimate individual genetic effects, from which polygenic scores can be derived. Designs capitalising on trio genomic data (including mother, father, and child) can be implemented to estimate familial genetic effects as well as gauge the presence of passive and/or active/evocative gene–environment correlations (Kong et al., 2018; Pingault, Barkhuizen, et al., 2021). These designs also offer new opportunities to better understand the role of parental risk factors in shaping the child's environment. For example, the ‘virtual parent’ design utilises trio data to construct polygenic scores comprising nontransmitted alleles (i.e. alleles not transmitted from parent to child). In turn, polygenic scores comprising nontransmitted alleles can be used to better understand the role of parental risk factors on child outcomes, independent of genetic transmission. For example, a polygenic score including nontransmitted alleles for depression can be used to assess the impact of exposure to parental depression. This design parallels the in vitro fertilisation design where the genetic relatedness between the parent and the child is broken when a donor gamete is implanted (Thapar & Rice, 2021). The absence of genetic relatedness precludes genetic confounding. Beyond polygenic scores, methods using genetic variants to better understand causality such as intergenerational Mendelian randomisation can also be implemented (Hwang, Davies, Warrington, & Evans, 2021; Pingault, Richmond, et al., 2021; Richmond et al., 2017; Zhang et al., 2015).

## Compounded challenges

Most polygenic score studies will be susceptible to multiple sources of bias, which, when combined, can make the interpretation of results challenging. Let’s take the example examined in Agnew‐Blais et al. (2022) of a plausible environmental risk factor, household chaos, predicting child ADHD symptoms. The child polygenic score for ADHD is associated with household chaos, suggesting the presence of genetic confounding. However, concluding that household chaos is an environmental factor for child ADHD after merely adjusting for the ADHD polygenic score is meaningless in the absence of a consideration of measurement error (Figure 1). As discussed above, careful sensitivity analyses using the polygenic score for the outcome to minimise collider bias in conjunction with heritability estimates can better answer this question (Figure 2) (Pingault, Rijsdijk, et al., 2021). Applying these methods shows that the association between household chaos and child ADHD is likely entirely accounted for by genetic confounding. In addition, the association between the child's polygenic score for ADHD and self‐reported household chaos itself cannot be readily interpreted. It can reflect perception bias, for example in the case of a hyperactive adolescent rating the household environment as chaotic (Figure 4). Comparing associations between the polygenic score and subjective versus more objective (e.g. external observer) measures of family chaos can help to assess the role of perception bias. Empirical findings show that the association between the polygenic score and the objective measure of household chaos appears larger than the association with subjective ratings, ruling out that the findings are entirely due to perception bias. Second, the association between the child polygenic score for ADHD and household chaos can reflect either evocative or passive gene–environment correlations (Figure 6). The use of polygenic scores based on trio genetic data can help identify the respective role of active/evocative versus passive *r_GE_* (e.g. the association between the child score and household chaos may disappear when controlling for parental polygenic scores, suggesting passive gene–environment correlation). Empirical findings show that adjusting for maternal polygenic scores does not eliminate the relationship between the child's polygenic score and household chaos, thereby suggesting evocative *r_GE_* (additional control for the father’s polygenic score would be required to confirm this finding). After careful examination, Agnew‐Blais et al. concluded that household chaos is unlikely to directly influence the development of child ADHD symptoms.

Importantly, the consequences of the biases discussed in this review are not necessarily negative, depending on the intended purpose of the analysis. For example, the fact that child polygenic scores potentially capture familial genetic effects and population stratification can be useful. Adjusting for such polygenic scores removes confounding beyond (strictly defined) genetic confounding. Such a polygenic score will also be useful when building a prediction model, where what counts is the strength of the association rather than understanding the underlying mechanisms.

## Conducting, reporting, and interpreting polygenic score studies

When conducting polygenic score studies, key sources of biases should be either addressed through analyses or discussed as limitations. A nonexhaustive list is presented in Table 2.

**Table 2:** Biases in polygenic score studies

Bias	Consequences	Possible solutions
Measurement error	Introduces bias in all multivariate models intending to estimate adjusted effects, mediation, and interaction effects.	Account explicitly for measurement error in the polygenic score and phenotypes when possible. Discuss biases arising from measurement errors.
Collider bias	Biases associations when adjusting for a collider such as a genetically influenced exposure.	Use the polygenic score for the outcome rather than the exposure in multivariate models to minimise collider bias.
Selection and attrition biases	Can generate spurious associations between polygenic scores and nongenetic predictors of attrition such as environmental variables.	Provide descriptive (e.g. testing whether polygenic scores are associated with attrition). Compare results in studies with low versus high attrition when available. Use appropriate methods to deal with attrition (e.g. imputation).
Perception bias	When only subjective measures are available: Generate false‐positive *r_GE_* and genetic confounding. Risk to over or underestimate the importance of risk factors.	Compare polygenic associations with subjective versus objective measures of risk. Discuss the respective aetiological roles of objective versus subjective risks.
Population stratification	Generates spurious associations between polygenic scores and phenotypes.	Adjust for principal components of ancestry along with other required technical variables. Rely on within‐family GWAS (e.g. within‐sibship) to better estimate individual genetic effects.
Assortative mating	Generates spurious associations between polygenic scores and phenotypes.	Implement within‐family GWAS (e.g. within‐sibship) to better estimate individual genetic effects.
Familial genetic effects	The association between a child's polygenic score and child phenotype does not only reflect individual genetic effects. The association between a child's polygenic score and the child's environment does not necessarily reflect active/evocative *r_GE_*	Implement within‐family analyses (e.g. trio design, to distinguish between individual and familial genetic effects).

As in other fields, triangulation of evidence is required, both within polygenic score studies, for example, comparing population and within‐family analyses (Howe et al., 2021; Selzam et al., 2019) or with other genetically informed methods (Pingault et al., 2018; Richmond & Smith, 2021; Smith, Richmond, & Pingault, 2021). For example, findings that polygenic scores for psychiatric disorders predict self‐harm have been followed by Mendelian randomisation analyses to assess causality (Lim et al., 2020). To assess whether an increased liability to ADHD leads to increased BMI, findings have been triangulated across developmentally sensitive within‐twin polygenic score analyses, Mendelian randomisation, and twin differences analyses (Liu et al., 2021). Findings using polygenic scores to estimate genetic nurture effects can also be discussed in view of evidence from genetic and nongenetic designs (Wang et al., 2021).

Biases included in Table 2 overlap with biases commonly encountered in epidemiological studies and those more specific to genetic studies. Conducting and interpreting polygenic score studies thus requires researchers in child psychology and psychiatry to be versed in both epidemiological and genetic methods or build on interdisciplinary collaborations.

## Supporting information


**Figure S1**. Representing relationships between variables.
**Figure S2**. Collider bias includes measurement errors.
**Appendix S1**. Direct genetic effect with measurement error (Figure 2).
**Appendix S2**. Exposure model (Figure 3).
**Appendix S3**. Collider bias (Figure S2).
