# Supplementary material for: Research Review: How to interpret associations between polygenic scores, environmental risks, and phenotypes
Source: J Child Psychol Psychiatry. 2022 Mar 28;63(10):1125–39. doi: 10.1111/jcpp.13607 (PMC9790749; doi:10.1111/jcpp.13607)
Supplement: Supplementary file 1 — Figure S1. Representing relationships between variables. Figure S2. Collider bias includes measurement errors. Appendix S1. Direct genetic effect with measurement error (Figure 2). Appendix S2. Exposure model (Figure 3). Appendix S3. Collider bias (Figure S2). [file JCPP-63-1125-s001.pdf]

Supporting Information

Research Review: How to interpret associations between polygenic scores, environmental risks, and phenotypes.

Jean-Baptiste Pingault, Andrea G. Allegrini, Tracy Odigie, Leonard Frach, Jessie R. Baldwin, Frühling Rijsdijk, Frank Dudbridge.

Table of contents:

General comments 1

Figure S1. Representing relationships between variables 2

Appendix S1. Direct genetic effect with measurement error (Figure 2) 3

Appendix S2. Exposure model (Figure 3) 5

Appendix S3. Collider bias (Figure S2) 13

General comments

In order to understand bias, derivations in the supplementary material express the beta from the fitted model as a function of the corresponding true parameter (i.e. beta from the underlying data-generating model). In each example, we follow the steps:

Step 1: Write true and fitted model equations
Step 2: In the fitted model, find fitted betas as a function of the observed correlations
Step 3: In the true model, find the observed correlations as a function of the true betas
Step 4: In equations from 2, replace observed correlations with their expressions derived from 3, thereby obtaining the fitted betas as a function of the true parameters, e.g. fitted beta = true beta + bias.

Notation throughout:
G^* = true additive genetic factor
X^* = true exposure
Y^* = true outcome
G = observed genetic effects
X = observed exposure
Y = observed outcome
l = measurement error (loading)
r = correlation
b = fitted beta
β = true beta
e = error in fitted models
ε = error in true models
V = variance
p_M = fitted proportion mediated
π_M = true proportion mediated

Derivations for the first two examples are presented in detail for readers new to structural equation modelling. The following variance-covariance rules are used throughout:

$$Var(X) = Cov(X, X)$$

$$Cov(aX, bY) = abCov(X, Y)$$

$$Cov(X, Y+Z) = Cov(X, Y) + Cov(X, Z)$$

We assume uncorrelated errors:

- Error terms of different variables are uncorrelated
- The error term of one variable is uncorrelated with other variables
- The error term of an outcome is unrelated to the predictor variable

However, the error term of a given variable (e.g. residuals of *Y* on *X*) remains correlated with the variable (e.g. *Y*) and cannot be ignored.

The structural equation models in Figure 1 and 2 include error terms and illustrate how error terms are handled, for example the absence of a double-headed arrow between ε_X and ε_Y encodes the assumption of uncorrelated errors, but the arrow from ε_{Y*} to $Y*$ encodes the association between the error term (or residual) and the corresponding variable.

Models are represented in manuscript's figures or supplementary figures.

Figure S1. Representing relationships between variables

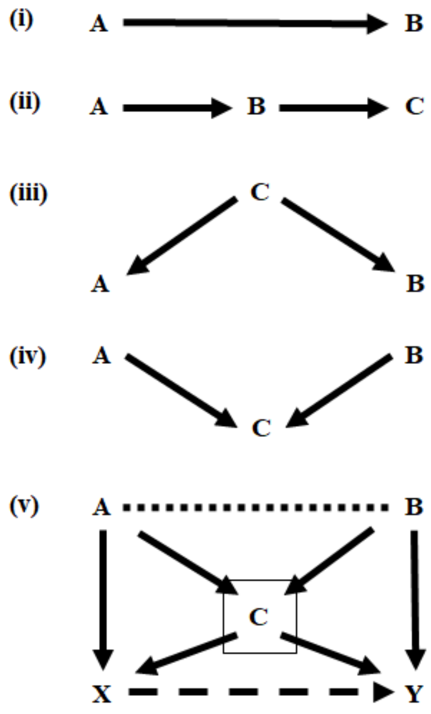

Figure 1 Caption. Figure S1 illustrates terms used throughout the article. The terms are borrowed from directed acyclic graphs (DAGs), that can be used to encode causal models and assumptions. In (i) the directed arrow encodes a causal effect of A on B. In (ii) the directed path goes from A to C via B. B is here a mediator, in the sense that the causal effect of A on C is happening indirectly via B. In (iii) C directly causes A and B. C is therefore a confounder of the association between A and B. The path between A and B via C is called a 'backdoor' path. A backdoor path creates an observed association between A and B even in the absence of a causal effect, represented by the absence of a directed arrow between A and B. In (iv) C is a collider as both arrows from A and B 'collide' in C. In this situation, the path is blocked in C. Contrary to the confo18under situation, there is no observed association between A and B. However, if C is adjusted for, this creates a spurious association between A and B. In (v) C is a confounder of X and Y and should therefore be adjusted for in order to retrieve the causal effect of X on Y. However, C is also a collider of A and B. Adjusting for C thus creates a spurious association between A and B, which introduces a backdoor path from X to Y via A and B.

Appendix S1. Direct genetic effect with measurement error (Figure 2)

Here the outcome is measured with error, but the polygenic score can also be conceived as a measure with error of true additive genetic effects from common SNPs. This is encoded in the Figure by the arrow from $G*$ to G, with the loading l_G.

Step 1. Model equations for the true and fitted model

Model equations for the true model:

$$Y^* = \beta_{G^* Y^*} G^* + \varepsilon_{Y^*}$$

$$Y = l_Y Y^* + \varepsilon_Y$$

$$G = l_G G^* + \varepsilon_G$$

$$G^* = G^*$$

Model equations for the fitted model:

$$Y = b_{GY} G + e_Y$$

$$G = G$$

Step 2. Find fitted betas as a function of the observed correlations for the fitted model

We have only two variables so the standardised regression estimate is equal to the observed correlation between the two variables.

$$b_{GY} = r_{GY}$$

Step 3. Find the observed correlations as a function of the true betas for the true model

$$\begin{aligned}
Cov(Y, G) &= Cov\left(l_Y Y^* + \varepsilon_Y, l_G G^* + \varepsilon_G\right) \\
&= l_Y l_G Cov(Y^*, G^*) + l_Y Cov\left(Y^*, \varepsilon_G\right) + l_G Cov\left(\varepsilon_Y, G^*\right) + Cov\left(\varepsilon_Y, \varepsilon_G\right) \\
&= l_Y l_G Cov\left(\beta_{G^* Y^*} G^* + \varepsilon_Y^*, G^*\right) + 0 + 0 + 0 \\
&= l_Y l_G \left[Cov\left(\beta_{G^* Y^*} G^*, G^*\right) + Cov\left(\varepsilon_Y^*, G^*\right)\right] \\
&= l_Y l_G Cov\left(\beta_{G^* Y^*} G^*, G^*\right) + 0 \\
&= l_Y l_G \beta_{G^* Y^*} V_{G^*}
\end{aligned}$$

Standardised version with variances equal 1:

$$r_{GY} = l_Y l_G \beta_{G^* Y^*}$$

Step 4. Replace observed correlations from step 2 with their expressions derived from step 3

We get:

$$b_{GY} = r_{GY} = l_Y l_G \beta_{G^* Y^*}$$

The fitted b_{GY} is thus equal to the true beta (β_{G*Y*}) attenuated by two loadings capturing measurement error for Y and G.

The **bias** is defined as the fitted effect minus the true effect:

$$Bias_{GY} = b_{GY} - \beta_{G*Y*} = l_Y l_G \beta_{G*Y*} - \beta_{G*Y*} = \beta_{G*Y*}\left(l_Y l_G - 1\right)$$

When $l_Y = l_G = 1$, there is no measurement error and thus no bias. Conversely, if the polygenic score or the outcome are pure noise (l_Y or $l_G = 0$), then the bias is total, equal to $-\beta_{G*Y*}$, leading to the observed association $b_{GY} = 0$.

Note that from above we have:

$$l_G^2 = \frac{r_{GY}^2}{l_Y^2 \beta_{G*Y*}^2}$$

Note that l_G^2 can be approximated by using SNP-heritability (h^2_{SNP}), assuming that

$$G* = G_{SNP}$$

If we further assume that h^2_{SNP} and the variance explained by the polygenic score are both affected in the same way by measurement error in the outcome (i.e. concretely, that means assuming equal measurement error for Y in the h^2_{SNP} study and in the polygenic score study), then we obtain the path from G_{SNP} to Y:

$$\sqrt{h^2_{SNP}} = l_Y \beta_{G*Y*}$$

and

$$l_G^2 = \frac{r_{GY}^2}{l_Y^2 \beta_{G*Y*}^2} = \frac{r_{GY}^2}{h^2_{SNP}}$$

Note that the equality $G* = G_{SNP}$ does not fully hold as SNP-heritability is not fitted based on all SNPs and SNP-heritability does not capture the total heritability. Hence the resulting reliability only refers to the reliability of the polygenic score as a measure of SNP-heritability rather than total heritability.

Appendix S2. Exposure model (Figure 3)

We consider the measurement error only in *G*, i.e. assuming *X** and *Y** are measured:

Step 1. Model equations for the true and fitted model

Model equations for the true model:

$$Y^* = \beta_{G^* Y^*} G^* + \beta_{X^* Y^*} X^* + \varepsilon_{Y^*}$$

$$X^* = \beta_{G^* X^*} G^* + \varepsilon_{X^*}$$

$$G = l_G G^* + \varepsilon_G$$

$$G^* = G^*$$

Variance required for the derivation:

$$V_{X^*} = Cov\left(\beta_{G^* X^*} G^* + \varepsilon_{X^*},\ \beta_{G^* X^*} G^* + \varepsilon_{X^*}\right) = \beta_{G^* X^*}{}^2 V_{G^*} + V_{\varepsilon_{X^*}}$$

Equations for the fitted model (in this case we keep *X** and *Y** assuming we have observed them, in discrepancy with the actual fitted model in Figure 3b):

$$Y^* = b_{GY^*} G + b_{X^* Y^*} X^* + e_{Y^*}$$

$$X^* = b_{GX^*} G + e_{X^*}$$

$$G = G$$

and the variance of *X** in the fitted model is:

$$V_{X^*} = b_{GX^*}{}^2 V_G + V_{e_{X^*}}$$

Step 2. Find fitted betas as a function of the observed correlations in the fitted model

Calculate correlations in the fitted model:

$$Cov(G,\ X^*) = Cov\left(G,\ b_{GX^*} G + e_{X^*}\right) = b_{GX^*} V_G$$

which when standardised corresponds to:

$$r_{GX^*} = b_{GX^*} \qquad (1)$$

and

$$Cov(G,\ Y^*) = Cov\left(G,\ b_{GY^*} G + b_{X^* Y^*} X^* + e_{Y^*}\right) = b_{GY^*} V_G + b_{X^* Y^*} Cov(G,\ X^*)$$

which when standardised corresponds to:

$$r_{GY^*} = b_{GY^*} + b_{X^* Y^*} b_{GX^*} \qquad (2)$$

and

$$\begin{aligned} Cov(X^*, Y^*) &= Cov\left(b_{GX^*}G + e_{X^*},\, b_{GY^*}G + b_{X^*Y^*}X^* + e_{Y^*}\right) \\ &= b_{GX^*}b_{GY^*}V_G + b_{GX^*}b_{X^*Y^*}Cov(G, X^*) + 0 + 0 + Cov\left(e_{X^*},\, b_{X^*Y^*}X^*\right) + 0 \\ &= b_{GX^*}b_{GY^*}V_G + b_{GX^*}{}^2 b_{X^*Y^*}V_G + b_{X^*Y^*}Cov\left(e_{X^*},\, b_{GX^*}G + e_{X^*}\right) \\ &= b_{GX^*}b_{GY^*}V_G + b_{GX^*}{}^2 b_{X^*Y^*}V_G + b_{X^*Y^*}\left[Cov\left(e_{X^*},\, b_{GX^*}G\right) + V_{e_{X^*}}\right] \\ &= b_{GX^*}b_{GY^*}V_G + b_{GX^*}{}^2 b_{X^*Y^*}V_G + b_{X^*Y^*}V_{e_{X^*}} \\ &= b_{GX^*}b_{GY^*}V_G + b_{X^*Y^*}\left(b_{GX^*}{}^2 V_G + V_{e_{X^*}}\right) \\ &= b_{GX^*}b_{GY^*}V_G + b_{X^*Y^*}V_{X^*} \end{aligned}$$

which when standardised corresponds to:

$$r_{X^*Y^*} = b_{GX^*}b_{GY^*} + b_{X^*Y^*} \qquad (3)$$

Next, we express betas as a function of observed correlations. From equation (2) we can retrieve:

$$b_{GY^*} = r_{GY^*} - b_{X^*Y^*}b_{GX^*}$$

and we can substitute this expression in equation (3), where we further include equation (1):

$$\begin{aligned} r_{X^*Y^*} &= b_{GX^*}\left(r_{GY^*} - b_{X^*Y^*}b_{GX^*}\right) + b_{X^*Y^*} \\ &= r_{GX^*}r_{GY^*} - r_{GX^*}{}^2 b_{X^*Y^*} + b_{X^*Y^*} \\ &= r_{GX^*}r_{GY^*} + b_{X^*Y^*}\left(1 - r_{GX^*}{}^2\right) \end{aligned}$$

So that:

$$b_{X^*Y^*} = \frac{r_{X^*Y^*} - r_{GX^*}r_{GY^*}}{1 - r_{GX^*}{}^2}$$

We can now turn to expressing b_{GY^*} as a function of observed correlations. From equations (1) and (3) we have:

$$b_{X^*Y^*} = r_{X^*Y^*} - r_{GX^*}b_{GY^*}$$

and we can substitute this in equation (2) to get:

$$
\begin{aligned}
b_{GY*} &= r_{GY*} - b_{X*Y*} r_{GX*} \\
&= r_{GY*} - \left(r_{X*Y*} - r_{GX*} b_{GY*} \right) r_{GX*} \\
&= r_{GY*} - r_{X*Y*} r_{GX*} + r_{GX*} r_{GX*} b_{GY*}
\end{aligned}
$$

Thus, we get:

$$
b_{GY*} = \frac{r_{GY*} - r_{X*Y*} r_{GX*}}{1 - r_{GX*}^{\ 2}}
$$

Step 3. Find the observed correlations as a function of the true betas for the true model

$$
\begin{aligned}
Cov(G, X*) &= Cov\left(l_G G* + \varepsilon_G, \beta_{G*X*} G* + \varepsilon_{X*} \right) \\
&= Cov\left(l_G G*, \beta_{G*X*} G* \right) + Cov\left(l_G G*, \varepsilon_{X*} \right) + Cov\left(\varepsilon_G, \beta_{G*X*} G* \right) + Cov\left(\varepsilon_G, \varepsilon_{X*} \right) \\
&= l_G \beta_{G*X*} V_{G*}
\end{aligned}
$$

which when standardised corresponds to:

$$
r_{GX*} = l_G \beta_{G*X*}
$$

And

$$
\begin{aligned}
Cov(Y*, G) &= Cov\left(\beta_{G*Y*} G* + \beta_{X*Y*} X* + \varepsilon_{Y*}, l_G G* + \varepsilon_G \right) \\
&= Cov\left(\beta_{G*Y*} G*, l_G G* \right) + Cov\left(\beta_{G*Y*} G*, \varepsilon_G \right) + Cov\left(\beta_{X*Y*} X*, l_G G* \right) + \\
&\quad Cov\left(\beta_{X*Y*} X*, \varepsilon_G \right) + Cov\left(\varepsilon_{Y*}, l_G G* \right) + Cov\left(\varepsilon_{Y*}, \varepsilon_G \right) \\
&= Cov\left(\beta_{G*Y*} G*, l_G G* \right) + 0 + Cov\left(\beta_{X*Y*} X*, l_G G* \right) + 0 + 0 + 0 \\
&= l_G \beta_{G*Y*} V_{G*} + l_G \beta_{X*Y*} Cov(X*, G*) \\
&= l_G \beta_{G*Y*} V_{G*} + l_G \beta_{X*Y*} Cov\left(\beta_{G*X*} G* + \varepsilon_{X*}, G* \right) \\
&= l_G \beta_{G*Y*} V_{G*} + l_G \beta_{X*Y*} \beta_{G*X*} V_{G*}
\end{aligned}
$$

which when standardised corresponds to:

$$r_{GY*} = l_G\left(\beta_{G*Y*} + \beta_{G*X*}\beta_{X*Y*}\right)$$

And

$$\begin{aligned}
Cov(X^*, Y^*) &= Cov\left(\beta_{G*X*}G^* + \varepsilon_{X*}, \beta_{G*Y*}G^* + \beta_{X*Y*}X^* + \varepsilon_{Y*}\right) \\
&= Cov\left(\beta_{G*X*}G^*, \beta_{G*Y*}G^*\right) + Cov\left(\beta_{G*X*}G^*, \beta_{X*Y*}X^*\right) + Cov\left(\beta_{G*X*}G^*, \varepsilon_{Y*}\right) + \\
&\quad Cov\left(\varepsilon_{X*}, \beta_{G*Y*}G^*\right) + Cov\left(\varepsilon_{X*}, \beta_{X*Y*}X^*\right) + Cov\left(\varepsilon_{X*}, \varepsilon_{Y*}\right) \\
&= Cov\left(\beta_{G*X*}G^*, \beta_{G*Y*}G^*\right) + Cov\left(\beta_{G*X*}G^*, \beta_{X*Y*}X^*\right) + 0 + \\
&\quad 0 + Cov\left(\varepsilon_{X*}, \beta_{X*Y*}X^*\right) + 0 \\
&= \beta_{G*X*}\beta_{G*Y*}V_{G*} + \beta_{G*X*}\beta_{X*Y*}Cov(G^*, X^*) + \beta_{X*Y*}Cov\left(\varepsilon_{X*}, X^*\right) \\
&= \beta_{G*X*}\beta_{G*Y*}V_{G*} + \beta_{G*X*}\beta_{X*Y*}Cov\left(G^*, \beta_{G*X*}G^* + \varepsilon_{X*}\right) + \\
&= \beta_{X*Y*}Cov\left(\varepsilon_{X*}, \beta_{G*X*}G^* + \varepsilon_{X*}\right) \\
&= \beta_{G*X*}\beta_{G*Y*}V_{G*} + \beta_{G*X*}{}^2\beta_{X*Y*}V_{G*} + \beta_{X*Y*}V_{\varepsilon_{X*}} \\
&= \beta_{G*X*}\beta_{G*Y*}V_{G*} + \beta_{X*Y*}V_{X*}
\end{aligned}$$

which when standardised corresponds to:

$$r_{X*Y*} = \beta_{G*X*}\beta_{G*Y*} + \beta_{X*Y*}$$

Step 4. Replace observed correlations from equation (2) with their expressions derived from equation (3)

We now can rewrite the fitted betas as:

$$b_{GX*} = r_{GX*} = l_G \beta_{G*X*}$$

and

$$\begin{aligned}
b_{X*Y*} &= \frac{r_{X*Y*} - r_{GX*} r_{GY*}}{1 - r_{GX*}^2} \\
&= \frac{\beta_{G*X*}\beta_{G*Y*} + \beta_{X*Y*} - l_G \beta_{G*X*} l_G \left(\beta_{G*Y*} + \beta_{G*X*}\beta_{X*Y*}\right)}{1 - l_G^2 \beta_{G*X*}^2} \\
&= \frac{\beta_{G*X*}\beta_{G*Y*} + \beta_{X*Y*} - l_G^2 \beta_{G*X*}\beta_{G*Y*} - l_G^2 \beta_{G*X*}\beta_{G*X*}\beta_{X*Y*}}{1 - l_G^2 \beta_{G*X*}^2} \\
&= \frac{\beta_{G*X*}\beta_{G*Y*}\left(1 - l_G^2\right) + \beta_{X*Y*}\left(1 - l_G^2 \beta_{G*X*}^2\right)}{1 - l_G^2 \beta_{G*X*}^2} \\
&= \beta_{X*Y*} + \frac{\beta_{G*X*}\beta_{G*Y*}\left(1 - l_G^2\right)}{1 - l_G^2 \beta_{G*X*}^2}
\end{aligned}$$

The fitted b_{X*Y*} is therefore the true β_{X*Y*} plus the true genetic confounding $\beta_{G*X*}\beta_{G*Y*}$ scaled by the measurement error of G $(1 - l_G^2)$. This makes sense as the fitted beta should be larger than the true beta as genetic confounding has not been adjusted entirely to the extent that G is a noisy measure of $G*$.

$$Bias_{X*Y*} = \beta_{X*Y*} + \frac{\beta_{G*X*}\beta_{G*Y*}\left(1 - l_G^2\right)}{1 - l_G^2 \beta_{G*X*}^2} - \beta_{X*Y*} = \frac{\beta_{G*X*}\beta_{G*Y*}\left(1 - l_G^2\right)}{1 - l_G^2 \beta_{G*X*}^2}$$

The bias can thus be quite large if reliability (l_G^2) is low. Lastly, we have:

$$b_{GY*} = \frac{r_{GY*} - r_{X*Y*} r_{GX*}}{1 - r_{GX*}^2}$$

$$= \frac{l_G\left(\beta_{G*Y*} + \beta_{G*X*}\beta_{X*Y*}\right) - l_G\beta_{G*X*}\left(\beta_{G*X*}\beta_{G*Y*} + \beta_{X*Y*}\right)}{1 - l_G^2\beta_{G*X*}^2}$$

$$= \frac{l_G\beta_{G*Y*} + l_G\beta_{G*X*}\beta_{X*Y*} - l_G\beta_{G*X*}^2\beta_{G*Y*} - l_G\beta_{G*X*}\beta_{X*Y*}}{1 - l_G^2\beta_{G*X*}^2}$$

$$= \frac{l_G\beta_{G*Y*} - l_G\beta_{G*X*}^2\beta_{G*Y*}}{1 - l_G^2\beta_{G*X*}^2}$$

$$= \frac{\beta_{G*Y*} l_G\left(1 - \beta_{G*X*}^2\right)}{1 - l_G^2\beta_{G*X*}^2}$$

with the corresponding bias:

$$Bias_{GY*} = b_{GY*} - \beta_{G*Y*}$$

$$= \frac{\beta_{G*Y*} l_G\left(1 - \beta_{G*X*}^2\right) - \beta_{G*Y*}\left(1 - l_G^2\beta_{G*X*}^2\right)}{1 - l_G^2\beta_{G*X*}^2}$$

$$= \frac{\beta_{G*Y*}\left[l_G\left(1 - \beta_{G*X*}^2\right) - \left(1 - l_G^2\beta_{G*X*}^2\right)\right]}{1 - l_G^2\beta_{G*X*}^2}$$

$$= \frac{\beta_{G*Y*}\left(l_G - l_G\beta_{G*X*}^2 - 1 + l_G^2\beta_{G*X*}^2\right)}{1 - l_G^2\beta_{G*X*}^2}$$

$$= \frac{\beta_{G*Y*}\left(l_G - 1 + l_G^2\beta_{G*X*}^2 - l_G\beta_{G*X*}^2\right)}{1 - l_G^2\beta_{G*X*}^2}$$

$$= \frac{\beta_{G*Y*}\left[l_G - 1 + l_G\beta_{G*X*}^2\left(l_G - 1\right)\right]}{1 - l_G^2\beta_{G*X*}^2}$$

$$= \frac{\beta_{G*Y*}\left(l_G - 1\right)\left(1 + l_G\beta_{G*X*}^2\right)}{1 - l_G^2\beta_{G*X*}^2}$$

We now turn to the mediated effect of genetic factors on *Y* via *X*. The true mediating effect is:

$$\beta_M = \beta_{G*X*}\beta_{X*Y*}$$

The fitted mediated effect b_M using the polygenic score is:

$$b_M = b_{GX*} b_{X*Y*} = l_G \beta_{G*X*} \left[\beta_{X*Y*} + \frac{\beta_{G*X*} \beta_{G*Y*} \left(1 - l_G^2\right)}{1 - l_G^2 \beta_{G*X*}^2} \right]$$

$$= l_G \beta_M + l_G \beta_{G*X*} \frac{\beta_{G*X*} \beta_{G*Y*} \left(1 - l_G^2\right)}{1 - l_G^2 \beta_{G*X*}^2}$$

$$= l_G \beta_M + l_G \beta_{G*X*} Bias_{X*Y*}$$

The fitted mediated model is therefore the true effect scaled by the loading of G ($l_G \beta_M$) but there is also an additional term corresponding to an additional "mediation" path via the $Bias_{X*Y*}$. This is because the fitted b_{X*Y*} is under-corrected for genetic confounding. This under correction exaggerates the path b_{X*Y*} which, in turn, exaggerates the fitted mediation.

In terms of proportion:
The true proportion mediated is:

$$\pi_M = \frac{\beta_M}{r_{G*Y*}}$$

and the fitted proportion mediated is:

$$p_M = \frac{b_{GX*} b_{X*Y*}}{r_{GY*}} = \frac{l_G \beta_{G*X*} \beta_{X*Y*} + l_G \beta_{G*X*} Bias_{X*Y*}}{l_G \left(\beta_{G*Y*} + \beta_{G*X*} \beta_{X*Y*} \right)}$$

$$= \frac{\beta_{G*X*} \beta_{X*Y*} + \beta_{G*X*} Bias_{X*Y*}}{\beta_{G*Y*} + \beta_{G*X*} \beta_{X*Y*}}$$

$$= \frac{\beta_{G*X*} \beta_{X*Y*} + \beta_{G*X*} Bias_{X*Y*}}{r_{G*Y*}}$$

$$= \frac{\beta_{G*X*} \beta_{X*Y*}}{r_{G*Y*}} + \frac{\beta_{G*X*} Bias_{X*Y*}}{r_{G*Y*}}$$

$$= \pi_M + \frac{r_{G*X*} Bias_{X*Y*}}{r_{G*Y*}}$$

$$= \pi_M + \frac{Bias_{X*Y*} r_{G*X*}}{r_{G*Y*}}$$

Note that the second term

$$\frac{Bias_{X^*Y^*} r_{G^*X^*}}{r_{G^*Y^*}}$$

is always positive when G is a polygenic score for Y as shown below. We have:

$$r_{G^*X^*} = \beta_{G^*X^*}$$

so the term can be rewritten as:

$$\frac{Bias_{X^*Y^*} r_{G^*X^*}}{r_{G^*Y^*}} = \frac{\beta_{G^*X^*}\beta_{G^*Y^*}\left(1-l_G^{\,2}\right) r_{G^*X^*}}{\left(1-l_G^{\,2}\beta_{G^*X^*}^{\;2}\right) r_{G^*Y^*}} = \frac{r_{G^*X^*}^{\;2}\beta_{G^*Y^*}\left(1-l_G^{\,2}\right)}{\left(1-l_G^{\,2}\beta_{G^*X^*}^{\;2}\right) r_{G^*Y^*}}$$

where $r_{G^*Y^*}$ is the square root of the heritability of Y, which is positive, and $\beta_{G^*Y^*}$ is the direct path, which is either 0 if the effect is totally mediated by X^* or positive in case of partial mediation. Both terms $(1 - l_G^2)$ and $(1 - l_G^2\beta_{G^*X^*}^2)$ are > 0 and < 1.

So when there is total mediation, there will be either no bias in case X^* is a total mediator of the true genetic effects (i.e. $\beta_{G^*Y^*} = 0$) or, which is much more likely, the proportion of the polygenic score effect on Y^* mediated by X^* will be overestimated compared to the true proportion of additive genetic effects on Y^* mediated by X^*.

Appendix S3. Collider bias (Figure S2)

Derivations are conducted as above but with less detail.

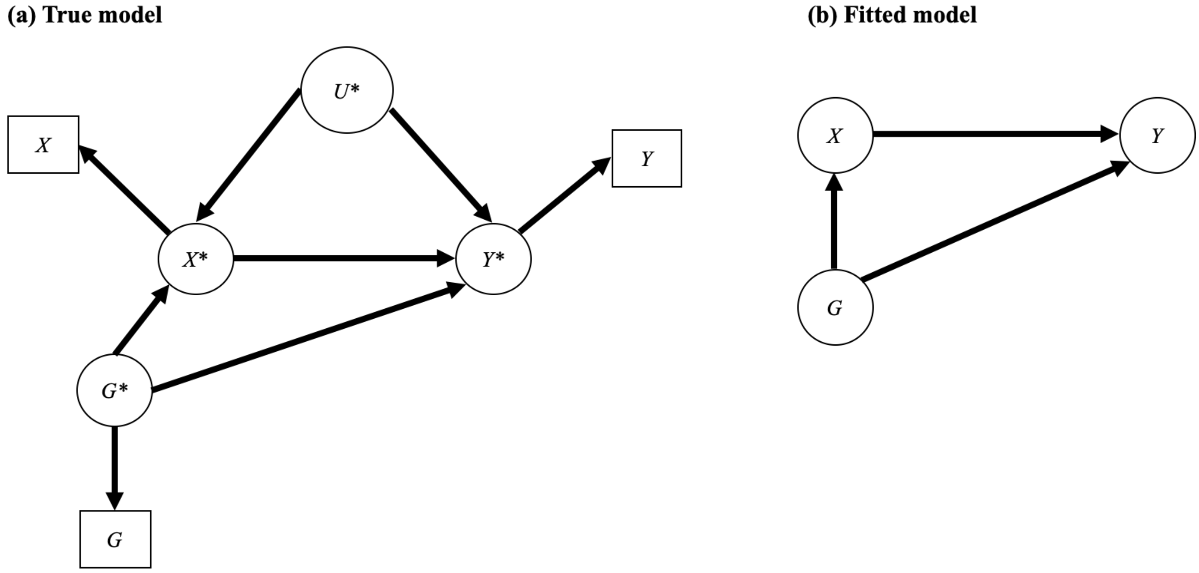

Figure S2. Collider bias including measurement errors

Step 1. Model equations for the true and fitted model

For the fitted model, we have the following equations:

$$Y = b_{GY}G + b_{XY}X + e_Y$$

$$X = b_{GX}G + e_X$$

$$G = G$$

and for the true model, we have the following equations:

$$Y^* = \beta_{G^*Y^*}G^* + \beta_{X^*Y^*}X^* + \beta_{U^*Y^*}U^* + \varepsilon_{Y^*}$$

$$X^* = \beta_{G^*X^*}G^* + \beta_{U^*X^*}U^* + \varepsilon_{X^*}$$

$$G^* = G^*$$

$$U^* = U^*$$

$$G = l_G G^* + \varepsilon_G$$

$$X = l_X X^* + \varepsilon_X$$

$$Y = l_Y Y^* + \varepsilon_Y$$

The required variance of X^* is:

$$V_{X^*} = \beta_{G^*X^*}{}^2 V_{G^*} + \beta_{U^*X^*}{}^2 V_{U^*} + V_{\varepsilon_{X^*}}$$

Step 2. Find fitted betas as a function of the observed correlations for the fitted model

The fitted model is analogous to the model derived for Figure 2, leading to:

$$b_{XY} = \frac{r_{XY} - r_{GX} r_{GY}}{1 - r_{GX}^2}$$

$$b_{GY} = \frac{r_{GY} - r_{XY} r_{GX}}{1 - r_{GX}^2}$$

Step 3. In the true model, find the observed correlations as a function of the true betas

We have:

$$Cov(G, X) = Cov\left(l_G G^* + \varepsilon_G, \; l_X X^* + \varepsilon_X \right) = l_G l_X Cov(G^*, X^*)$$

and:

$$\begin{aligned} Cov(G^*, X^*) &= Cov\left(G^*, \beta_{G^* X^*} G^* + \beta_{U^* X^*} U^* + \varepsilon_{X^*} \right) \\ &= Cov\left(G^*, \beta_{G^* X^*} G^* \right) + Cov\left(G^*, \beta_{U^* X^*} U^* \right) + Cov\left(G^*, \varepsilon_{X^*} \right) \\ &= \beta_{G^* X^*} V_{G^*} + \beta_{U^* X^*} Cov(G^*, U^*) \end{aligned}$$

If, as encoded in the figure, U* represents non-genetic unknown confounders, then:

$$Cov(G^*, U^*) = 0$$

we have:

$$Cov(G^*, X^*) = \beta_{G^* X^*} V_{G^*}$$

Thus, we can finally derive:

$$Cov(G, X) = l_G l_X \left(\beta_{G^* X^*} V_{G^*} \right)$$

which when standardised corresponds to:

$$r_{GX} = l_G l_X \beta_{G^* X^*}$$

and

$$\begin{aligned}
Cov(X, Y) &= Cov\left(l_X X^* + \varepsilon_X, l_Y Y^* + \varepsilon_Y\right) \\
&= l_X l_Y Cov(X^*, Y^*) + 0 + 0 + 0 \\
&= l_X l_Y Cov\left(\beta_{G^*X^*} G^* + \beta_{U^*X^*} U^* + \varepsilon_{X^*}, \beta_{G^*Y^*} G^* + \beta_{X^*Y^*} X^* + \beta_{U^*Y^*} U^* + \varepsilon_{Y^*}\right) \\
&= l_X l_Y [\beta_{G^*X^*}\beta_{G^*Y^*} V_{G^*} + \beta_{G^*X^*}\beta_{X^*Y^*} Cov(G^*, X^*) + \beta_{U^*X^*}\beta_{X^*Y^*} Cov(U^*, X^*) + \\
&\quad \beta_{U^*X^*}\beta_{U^*Y^*} V_{U^*} + \beta_{X^*Y^*} Cov\left(\varepsilon_{X^*}, X^*\right)] \\
&= l_X l_Y [\beta_{G^*X^*}\beta_{G^*Y^*} V_{G^*} + \beta_{G^*X^*}\beta_{X^*Y^*}\beta_{G^*X^*} V_{G^*} + \\
&\quad \beta_{U^*X^*}\beta_{X^*Y^*} Cov\left(U^*, \beta_{G^*X^*} G^* + \beta_{U^*X^*} U^* + \varepsilon_{X^*}\right) + \\
&\quad \beta_{U^*X^*}\beta_{U^*Y^*} V_{U^*} + \beta_{X^*Y^*} Cov\left(\varepsilon_{X^*}, \beta_{G^*X^*} G^* + \beta_{U^*X^*} U^* + \varepsilon_{X^*}\right)] \\
&= l_X l_Y [\beta_{G^*X^*}\beta_{G^*Y^*} V_{G^*} + \beta_{G^*X^*}{}^2 \beta_{X^*Y^*} V_{G^*} + \beta_{U^*X^*}{}^2 \beta_{X^*Y^*} V_{U^*} + \\
&\quad \beta_{U^*X^*}\beta_{U^*Y^*} V_{U^*} + \beta_{X^*Y^*} V\varepsilon_{X^*}] \\
&= l_X l_Y [\beta_{G^*X^*}\beta_{G^*Y^*} V_{G^*} + \beta_{U^*X^*}\beta_{U^*Y^*} V_{U^*} + \beta_{X^*Y^*}\left(\beta_{G^*X^*}{}^2 V_{G^*} + \beta_{U^*X^*}{}^2 V_{U^*} + V_{\varepsilon_{X^*}}\right)] \\
&= l_X l_Y \left(\beta_{G^*X^*}\beta_{G^*Y^*} V_{G^*} + \beta_{U^*X^*}\beta_{U^*Y^*} V_{U^*} + \beta_{X^*Y^*} V_{X^*}\right)
\end{aligned}$$

which when standardised corresponds to:

$$r_{XY} = l_X l_Y \left(\beta_{G^*X^*}\beta_{G^*Y^*} + \beta_{U^*X^*}\beta_{U^*Y^*} + \beta_{X^*Y^*}\right)$$

and

$$\begin{aligned}
Cov(G, Y) &= Cov\left(l_G G^* + \varepsilon_G, l_Y Y^* + \varepsilon_Y\right) = l_G l_Y Cov(G^*, Y^*) \\
&= l_G l_Y Cov\left(G^*, \beta_{G^*Y^*} G^* + \beta_{X^*Y^*} X^* + \beta_{U^*Y^*} U^* + \varepsilon_{Y^*}\right) \\
&= l_G l_Y \left[\beta_{G^*Y^*} V_{G^*} + \beta_{X^*Y^*} Cov(G^*, X^*)\right] \\
&= l_G l_Y \left(\beta_{G^*Y^*} V_{G^*} + \beta_{X^*Y^*}\beta_{G^*X^*} V_{G^*}\right)
\end{aligned}$$

which when standardised corresponds to:

$$r_{GY} = l_G l_Y \left(\beta_{G^*Y^*} + \beta_{X^*Y^*}\beta_{G^*X^*}\right)$$

Step 4. Replace observed correlations from step 2 with the corresponding expressions derived from step 3

We now can rewrite the fitted correlations as:

$$
\begin{aligned}
b_{XY} &= \frac{r_{XY} - r_{GX} r_{GY}}{1 - r_{GX}^2} \\
&= \frac{l_X l_Y\left(\beta_{G*X*}\beta_{G*Y*} + \beta_{U*X*}\beta_{U*Y*} + \beta_{X*Y*}\right) - l_G l_X \beta_{G*X*} l_G l_Y\left(\beta_{G*Y*} + \beta_{X*Y*}\beta_{G*X*}\right)}{1 - \left(l_G l_X \beta_{G*X*}\right)^2} \\
&= \frac{l_X l_Y\left(\beta_{G*X*}\beta_{G*Y*} + \beta_{U*X*}\beta_{U*Y*} + \beta_{X*Y*} - l_G^2 \beta_{G*X*}\beta_{G*Y*} - \beta_{X*Y*} l_G^2 \beta_{G*X*}^2\right)}{1 - \left(l_G l_X \beta_{G*X*}\right)^2} \\
&= \frac{l_X l_Y\left[\beta_{X*Y*}\left(1 - l_G^2 \beta_{G*X*}^2\right) + \beta_{U*X*}\beta_{U*Y*} + \beta_{G*X*}\beta_{G*Y*}\left(1 - l_G^2\right)\right]}{1 - \left(l_G l_X \beta_{G*X*}\right)^2}
\end{aligned}
$$

If we now assume no measurement error $l_G = l_X = l_Y = 1$, we find the expression first presented in the manuscript for collider bias, i.e.:

$$
b_{X*Y*} = \beta_{X*Y*} + \frac{\beta_{U*X*}\beta_{U*Y*}}{1 - \beta_{G*X*}^2}
$$

If we assume only perfect measurement of *X** and *Y**, which means that b_{XY} becomes b_{X*Y*}, we have $l_X = l_Y = 1$, and

$$
\begin{aligned}
b_{X*Y*} &= \frac{\beta_{X*Y*}\left(1 - l_G^2 \beta_{G*X*}^2\right) + \beta_{U*X*}\beta_{U*Y*} + \beta_{G*X*}\beta_{G*Y*}\left(1 - l_G^2\right)}{1 - \left(l_G \beta_{G*X*}\right)^2} \\
&= \beta_{X*Y*} + \frac{\beta_{U*X*}\beta_{U*Y*} + \beta_{G*X*}\beta_{G*Y*}\left(1 - l_G^2\right)}{1 - \left(l_G \beta_{G*X*}\right)^2}
\end{aligned}
$$

We see that when the polygenic score imperfectly captures the heritability, the fitted b_{XY} is equal to the true β_{X*Y*} plus additional bias terms that are more complex. To explain them, let's first assume that the polygenic score does not measure anything, $l_G = 0$, we have:

$$
b_{X*Y*} = \beta_{X*Y*} + \beta_{U*X*}\beta_{U*Y*} + \beta_{G*X*}\beta_{G*Y*}
$$

That is, the fitted beta is equal to the true beta, plus the confounding effect via U^* and genetic confounding, which is reintroduced in full as the polygenic score does not adjust for genetic confounding. The collider bias disappears as, in effect, there is no adjustment.

If the polygenic score measures some of the liability, then $0 < l_G < 1$, and we get two terms adding bias to $\beta_{X^*Y^*}$. First:

$$\frac{\beta_{U^*X^*}\beta_{U^*Y^*}}{1-\left(l_G\beta_{G^*X^*}\right)^2}$$

Here, a higher l_G in the denominator leads to a smaller denominator, which increases the collider bias via U. This is important as it means that the bias is high when G^* explains more of X^* (e.g. highly heritable X^*) and that better polygenic scores will better capture the association between G^* and X^* and increase collider bias.

The second term is

$$\frac{\beta_{G^*X^*}\beta_{G^*Y^*}\left(1-l_G^2\right)}{1-\left(l_G\beta_{G^*X^*}\right)^2}$$

This means that when $l_G < 1$, genetic confounding is reintroduced to some extent. An increasing l_G will reduce genetic confounding, and $l_G = 1$ will eliminate genetic confounding.

Overall, more accurate polygenic scores will thus remove confounding more efficiently but also increase collider bias.

We further have:

$$b_{GY}=\frac{r_{GY}-r_{XY}r_{GX}}{1-r_{GX}^2}$$

$$=\frac{l_Gl_Y\left(\beta_{G^*Y^*}+\beta_{X^*Y^*}\beta_{G^*X^*}\right)-l_Gl_X\beta_{G^*X^*}l_Xl_Y\left(\beta_{G^*X^*}\beta_{G^*Y^*}+\beta_{U^*X^*}\beta_{U^*Y^*}+\beta_{X^*Y^*}\right)}{1-\left(l_G\beta_{G^*X^*}\right)^2}$$

$$=\frac{l_Gl_Y\left(\beta_{G^*Y^*}+\beta_{X^*Y^*}\beta_{G^*X^*}-l_X^2\beta_{G^*X^*}\beta_{G^*X^*}\beta_{G^*Y^*}-l_X^2\beta_{G^*X^*}\beta_{U^*X^*}\beta_{U^*Y^*}-l_X^2\beta_{G^*X^*}\beta_{X^*Y^*}\right)}{1-\left(l_G\beta_{G^*X^*}\right)^2}$$

$$=\frac{l_Gl_Y\left[\beta_{G^*Y^*}\left(1-l_X^2\beta_{G^*X^*}^2\right)+\beta_{X^*Y^*}\beta_{G^*X^*}\left(1-l_X^2\right)-l_X^2\beta_{G^*X^*}\beta_{U^*X^*}\beta_{U^*Y^*}\right]}{1-\left(l_G\beta_{G^*X^*}\right)^2}$$

Note that if measurement is perfect, $l_G = l_Y = l_X = 1$ the expression simplifies to the expression for $b_{G^*Y^*}$ presented in the manuscript. Here, if $l_G = 0$ meaning that the polygenic score is entirely unreliable, then the entire expression is null, i.e. $b_{GY} = 0$, which is logical as the polygenic score is not associated with Y. As l_G increases the numerator increases, and the denominator decreases, leading to an increased b_{GY}

Measurement error in the outcome decreases b_{GY}. Note the importance of measurement error in the exposure l_X^2. If the measurement is not perfect, i.e., $l_X < 1$, there is an additional bias term

$$\beta_{X^*Y^*}\beta_{G^*X^*}\left(1 - l_X^2\right)$$

which arises because the effect of X^* is not entirely adjusted for. This means that even if genetic effects on Y are entirely explained by X^*, there will be a residual correlation between the polygenic score and the outcome. The third term corresponds to the collider bias via U^*:

$$l_X^2\beta_{G^*X^*}\beta_{U^*X^*}\beta_{U^*Y^*}$$

This term is also scaled by l_X^2, meaning that measurement error in the exposure weakens the collider effect. Measurement error in different variables can have effects of opposite directions on b_{GY} making it not straightforward to assess whether b_{GY} will be over or underestimated. For example, assuming all betas are positive, increased measurement error in G (lower l_G) will decrease b_{GY}, whereas increased measurement error in X (lower l_X) will increase b_{GY}.
